# The roles of pleiotropy and close linkage as revealed by association mapping of yield and correlated traits of wheat (*Triticum aestivum* L.)

**DOI:** 10.1093/jxb/erx214

**Published:** 2017-07-21

**Authors:** Albert W Schulthess, Jochen C Reif, Jie Ling, Jörg Plieske, Sonja Kollers, Erhard Ebmeyer, Viktor Korzun, Odile Argillier, Gunther Stiewe, Martin W Ganal, Marion S Röder, Yong Jiang

**Affiliations:** 1 Department of Breeding Research, Leibniz Institute of Plant Genetics and Crop Plant Research (IPK), Gatersleben, Germany; 2 TraitGenetics GmbH, Gatersleben, Germany; 3 KWS LOCHOW GmbH, Bergen, Germany; 4 Syngenta France S.A.S., Orgerus, France; 5 Syngenta Seeds GmbH, Bad Salzuflen, Germany

**Keywords:** Association, correlation, linkage, multivariate, pleiotropy, simulation, wheat, yield

## Abstract

Grain yield (GY) of bread wheat (*Triticum aestivum* L.) is quantitatively inherited. Correlated GY-syndrome traits such as plant height (PH), heading date (HD), thousand grain weight (TGW), test weight (TW), grains per ear (GPE), and ear weight (EW) influence GY. Most quantitative genetics studies assessed the multiple-trait (MT) complex of GY-syndrome using single-trait approaches, and little is known about its underlying pleiotropic architecture. We investigated the pleiotropic architecture of wheat GY-syndrome through MT association mapping (MT-GWAS) using 372 varieties phenotyped in up to eight environments and genotyped with 18 832 single nucleotide polymorphisms plus 24 polymorphic functional markers. MT-GWAS revealed a total of 345 significant markers spread genome wide, representing 8, 40, 11, 40, 34, and 35 effective GY–PH, GY–HD, GY–TGW, GY–TW, GY–GPE, and GY–EW associations, respectively. Among them, pleiotropic roles of *Rht-B1* and *TaGW2-6B* loci were corroborated. Only one marker presented simultaneous associations for three traits (i.e. GY–TGW–TW). Close linkage was difficult to differentiate from pleiotropy; thus, the pleiotropic architecture of GY-syndrome was dissected more as a cause of pleiotropy rather than close linkage. Simulations showed that minor allele frequencies, along with sizes and distances between quantitative trait loci for two traits, influenced the ability to distinguish close linkage from pleiotropy.

## Teaser

Multiple-trait approaches dissected the pleiotropic architecture of wheat yield and correlated traits more as a function of pleiotropy rather than close linkage. Factors affecting yield were investigated using simulations.

## Introduction

Grain yield (GY) of bread wheat (*Triticum aestivum* L.) has been continuously increased due to the interactions between technological advances in agronomical practices and genetic improvement (Borlaug, 1968; Austin *et al.*, 1980; Brancourt-Hulmel *et al.*, 2003; Laidig *et al.*, 2014). GY is a quantitatively inherited trait, and a substantial part of its variation is attributed to the environmental component and its interaction with genotypic variation (e.g. Crossa *et al.*, 2007; Kuchel *et al.*, 2007; Rattey *et al.*, 2009; Reif *et al.*, 2011). The genetic architecture of GY is very complex, and linkage (Huang *et al.*, 2006; Kuchel *et al.*, 2007; Kumar *et al.*, 2007; Cuthbert *et al.*, 2008; Bennett *et al.*, 2012; Simmonds *et al.*, 2014; Zhang *et al.*, 2014) and genome-wide association studies (GWAS; Crossa *et al.*, 2007; Neumann *et al.*, 2011; Reif *et al.*, 2011; Bordes *et al.*, 2014; Sukumaran *et al.*, 2015) have mostly reported minor quantitative trait loci (QTLs) influencing GY across the 21 chromosomes of bread wheat. Additionally, several loci underlying GY interact epistatically (e.g. Crossa *et al.*, 2007; Kumar *et al.*, 2007; Reif *et al.*, 2011; Zhang *et al.*, 2014), which can be explained by component traits that multiplicatively determine GY. These component traits include crop plant density, ears per plant, grains per ear (GPE), thousand grains weight (TGW), among others. Also, morphophysiological traits such as plant height (PH), heading date (HD), grain filling period length, plant sink:source ratios, and photosynthetic rate have direct or indirect effects on GY (Slafer *et al.*, 1996). We denote this group of traits influencing GY hereafter as GY-syndrome traits. Higher plot-based heritabilities have been reported for GY-syndrome traits than for GY itself (e.g. Rattey *et al.*, 2009; Reif *et al.*, 2011; Sukumaran *et al.*, 2015). Thus, wheat breeders are interested in GY-syndrome traits because of their possible use in indirect selection, potentially improving GY selection gain and accuracy (Falconer and Mackay, 1996).

Most quantitative genetic studies of the GY-syndrome in wheat have so far relied on single-trait (ST) methods (Huang *et al.*, 2006; Kuchel *et al.*, 2007; Cuthbert *et al.*, 2008; Neumann *et al.*, 2011; Bennett *et al.*, 2012; Bordes *et al.*, 2014; Zhang *et al.*, 2014; Sukumaran *et al.*, 2015). Nevertheless, ST approaches ignore the information from associations among traits. In contrast, this information is considered in multiple-trait (MT) models (Henderson and Quaas, 1976). Simulation and theoretical studies have shown the advantages of MT over ST models. For instance, a more precise estimation of QTL effects, an increased power of QTL detection, and a lower rate of false-positive MT associations are expected for MT approaches (Jiang and Zeng, 1995; Almasy *et al.*, 1997; Allison *et al.*, 1998; Mangin *et al.*, 1998; Williams *et al.*, 1999; Calinski *et al.*, 2000; Knott and Haley, 2000; Cheng *et al.*, 2013). In consequence, the interest in MT methods has markedly increased over the last years in the field of linkage studies (Lebreton *et al.*, 1998; Mangin *et al.*, 1998; Calinski *et al.*, 2000; Kumar *et al.*, 2007; Malosetti *et al.*, 2008; Deng *et al.*, 2011; Balestre *et al.*, 2012; Cheng *et al.*, 2013; Simmonds *et al.*, 2014) and, more recently, in the context of GWAS (Stich *et al.*, 2008; Jaiswal *et al.*, 2016; Thoen *et al.*, 2017).

MT methods can formally test if the co-location of QTLs for more than one trait is due to pleiotropy (i.e. a single genetic factor simultaneously controls several traits) or because of close linkage between more than one genetic factor influencing each trait separately (Jiang and Zeng, 1995; Almasy *et al.*, 1997; Williams *et al.*, 1999; Knott and Haley, 2000; Calinski *et al.*, 2000; Varona *et al.*, 2004; Da Costa E Silva *et al.*, 2012). Pleiotropy and linkage are the basis of genetic correlations (Falconer and Mackay, 1996), and the ability to differentiate between them will determine the optimum breeding strategy (Chen and Lübberstedt, 2010). For instance, if undesired trait correlations are caused by linkage, breeders have to put effort into breaking this correlation by means of recombination. Some linkage studies aiming to disentangle linkage from pleiotropy in crop plants can be found, for instance on GY and GY-syndrome traits in maize (Calinski *et al.*, 2000; Balestre *et al.*, 2012) and on average ear and leaf lengths under saline stress in wheat (Lebreton *et al.*, 1998). Nevertheless, to the best of our knowledge, this kind of study is lacking so far in the context of GWAS in crop plants.

The main goal of our study was to dissect the pleiotropic architecture of GY-syndrome by means of GWAS using MT statistical approaches in a bread wheat population of varieties adapted to European environments. The specific objectives were: first, to find genomic regions simultaneously associated with GY and GY-syndrome traits using MT-GWAS; secondly, to elucidate if the existence of these regions is a consequence of pleiotropy or due to closely linked non-pleiotropic QTLs; and, thirdly, to understand by means of simulations some factors that could have driven the ability to distinguish between these two phenomena in the current study. Our findings bring a better understanding of the pleiotropic architecture of GY-syndrome and its implications in applied MT wheat breeding.

## Materials and methods

### Plant material and phenotypic data

Our study is based on the GABI-WHEAT population which is composed of 358 European winter plus 14 spring wheat varieties. A detailed description of the varieties can be found in Kollers *et al.* (2013*a*). The GABI-WHEAT population was tested in up to eight environments throughout Germany (Seligenstadt 2009 and 2010, Wohlde 2009 and 2010) and France (Andelu 2009 and 2010, Saultain and Janville 2010). The experimental design for field trials was an alpha-lattice design with two replications. Plot sizes ranged from 5 m^2^ to 6.8 m^2^. Sowing rates and dates were according to local agricultural practices. PH (cm), HD (days since 1 January), and TGW (g) data from these experiments were already available from past studies (Zanke *et al.*, 2014*a*, *b*, 2015). Moreover, a sample of 10 ears was taken from each plot and then used to calculate GPE and EW (g). Plots were combine-harvested and grains were dried to reach 14% humidity. Later, GY was calculated in Mg ha^−1^. TW was measured from a clean grain sample using a 250 ml cylinder and subsequently expressed in kg hl^−1^. After a quality check, data for GY, PH, and HD remained available for all environments, while TGW and TW were unavailable for Saultain, and only one of two replicates was considered for these two traits in Andelu 2010 and Janville 2010. GPE was available for Andelu 2010 and Wohlde 2009, whereas EW from Wohlde 2009 and 2010 was taken into consideration for further phenotypic analyses.

### Molecular data

In a previous study (Zanke *et al.*, 2014*a*), the GABI-WHEAT population was genotyped by using a 90K Infinium single nucleotide polymorphism (SNP) chip (Wang *et al.*, 2014). Quality control of SNP markers was assessed as stated by Jiang *et al.* (2015), making a total of 18 832 SNP markers available for GWAS. In addition, the GABI-WHEAT population was genotyped using a set of 24 functional markers (Kollers *et al.*, 2013*a*, *b*; Zanke *et al.*, 2015) associated in past studies with agronomic traits, disease resistance, or grain quality (Supplementary Table S1 at *JXB* online). Missing values for functional markers with <10% missing data were imputed according to allele frequencies.

### Phenotypic analyses

Best linear unbiased estimators (BLUEs) and variance components were computed based on an unweighted two-stage univariate mixed model analysis approach (Möhring and Piepho, 2009). First, BLUEs of genotypes were separately computed for each trait-by-environment combination by considering the following model:

Trait~μ+Genotypes+Replicates+Blocks(Replicates)+Error (1)

where µ (i.e. the common mean), and genotypes were considered as fixed factors, whereas replicates, blocks nested within replicates, and error effects were considered as random. After this stage, BLUEs of all environments and unreplicated data were combined together. During the second stage, BLUEs of genotypes across environments for each single trait were computed using the following model:

Trait~μ+Genotypes+Environments+Error (2)

where µ and genotype effects were assumed as fixed factors, whereas environments and error terms were considered as random. In parallel, a model considering genotypes as random factor was fitted for estimation of variance components during the first and second stages. The genotypic variance (σ^2^_G_) was estimated during the second step, whereas the error (σ^2^_e_) and genotype×environment interaction (σ^2^_G×E_) variance components are mixed at this stage. Therefore, an error variance (σ^2^_Error_), namely the average of σ^2^_e_ in single environments with replications, was derived during the first step and subsequently subtracted from σ^2^_e_ during the second step for σ^2^_G×E_ estimation. Estimates of variance components were later considered for the computation of heritability (*h*^2^) on a plot basis as: $h_{Plot}^{2}=\frac{\sigma_{G}^{2}}{\sigma_{G}^{2}+\sigma_{G\times E}^{2}+\sigma_{Error}^{2}}$ . In addition, heritabilities were estimated on an entry-mean basis as presented by Piepho and Möhring (2007): $h_{Entry}^{2}=\frac{\sigma_{G}^{2}}{\sigma_{G}^{2}+\frac{\sigma_{G\times E}^{2}}{No.\;Env.}+\frac{\sigma_{Error}^{2}}{No.Rep.\times No.Env.}}$ , where No.Env. and No.Rep. denote the number of environments and the number of replications, respectively. Phenotypic correlations among the different traits considered in this study were calculated as the Pearson product–moment correlation coefficient between genotypic BLUEs of different traits across environments. Significance of phenotypic correlations was assessed by a *t*-test.

For the estimation of genetic correlations among traits, we considered an MT model according to Henderson and Quaas (1976):

$$y_{kji}=\mu_{k}+l_{kj}+g_{ki}+e_{kji}$$ (3)

where *y*_*kji*_ is the phenotype of the *i*th genotype at the *j*th environment for the *k*th trait (i.e. each ST BLUE or unreplicated data after the first stage of phenotypic analysis), μ _*k*_ corresponds to the overall mean of the *k*th trait, *l*_*kj*_ denotes the effect of the *j*th environment on the *k*th trait, *g*_*ki*_ represents the breeding value of the *i*th genotype for the *k*th trait, and *e*_*kji*_ is the error term for the *y*_*kji*_ observation according to the model. Terms μ _*k*_ and *l*_*kj*_ were designated as fixed, while *g*_*ki*_ and *e*_*kji*_ were considered random. In matrix nomenclature, the random terms *g*_*ki*_ and *e*_*kji*_ are assumed to be normally distributed in the way ***g***~*N*(0_(*t*×*n*)×1_, **G**) and **e**~*N*(0_*N*×1_, **R**), respectively, where 0_(*t*×*n*)×1_ and 0_*N*×1_ are null vectors of length *t*×*n* and *N*, respectively, with *t* and *n* being the number of traits and genotypes, respectively, whereas *N*=*t*×No.Env.×*n*. In addition, **G** denotes the variance–covariance structure between the *t*×*n* MT breeding values included within ***g***, while **R** represents the variance–covariance matrix for the *N* error terms contained in **e**. Provided that ***Y*** is the vector containing the *N* different *y*_*kji*_ values and that these elements are conveniently sorted so that genotypes and environments are nested within traits, **G** and **R** can be further decomposed as **G**=**G**_**0**_⊗**A** and **R**=**R**_**0**_⊗**I**, respectively, where **G**_**0**_ is the *t*×*t* additive genetic variance–covariance matrix among traits, ⊗ denotes the Kronecker product operator between matrices (Searle, 2006), **A** represents a relationship matrix between genotypes, **R**_**0**_ corresponds to the *t*×*t* error variance–covariance matrix among traits, and **I** is an identity matrix of order No.Env.×*n*. The **A** matrix was estimated by 2×(**J–RD**), where **J** denotes an *n*×*n* matrix whose every element is 1 and **RD** is a matrix containing the Rogers’ distances (Rogers, 1972) among *n* genotypes previously calculated from the SNP profiles in Jiang *et al.* (2015). As shown by Melchinger *et al.* (1991), these measurements of genetic similarity between homozygous inbred lines are, under simplifying assumptions, linearly related to the Mallecots’ (1948) coefficient of co-ancestry calculated from pedigree records. The pre-specification of the **A** matrix allowed the estimation of **G**_**0**_ by means of the restricted maximum likelihood algorithm. During analyses, *t* was fixed to 2 so that a bivariate model was fitted for each pair of traits. Denoting each element of **G**_**0**_ as *s*_*kk*'_, the genetic correlation between any pair of traits *k* and *k*' was calculated as $\frac{s_{kk^{'}}}{\sqrt{s_{kk}\times \;s_{k^{'}k^{'}}}}$ . Significance of *s*_*kk*'_ was tested by a likelihood ratio (LR) test comparing a model in which *s*_*kk*'_=0 with a model without this restriction. The LR under *H*_0_ (*s*_*kk*'_=0) follows a χ ^2^_1_ distribution and its significance was interpreted as evidence for a significant genetic correlation.

### Multiple-trait genome-wide association mapping

We firstly considered a bivariate GWAS approach for each of the six GY plus one GY-syndrome trait combinations following the suggestion of Stich *et al.* (2008). In principle, the model underlying this approach is an extension of Equation 3, allowing now the inclusion of a marker with pleiotropic effects on *k* traits. Then, the MT-GWAS model corresponds to:

$$y_{kji}=\mu_{k}+\alpha_{k}\times m_{i}+g_{ki}+l_{kj}+{\left(\alpha l\right)}_{kj}\times m_{i}+e_{kji}$$ (4)

where α _*k*_ represents the effect of a particular marker on the *k*th trait, *m*_*i*_ is a scalar taking values 0, 1, or 2 for individuals homozygous for the most frequent allele, heterozygous and homozygous for the second allele at the tested marker, respectively, whereas (α *l*)_*kj*_ denotes the interaction of this marker with the *j*th environment for the *k*th trait. Both, α _*k*_ and (α *l*)_*kj*_ were assumed as fixed effects within the linear mixed model. In the case of multi-allelic functional markers, we treated each allele as a bi-allelic marker for simplicity. The genetic diversity of the GABI-WHEAT population has been extensively studied using different molecular marker platforms (Kollers *et al.*, 2013*a*; Jiang *et al.*, 2015; Zanke *et al.*, 2015), and it was jointly concluded that there is no recognizable population structure in it. Therefore, only family structure correction using the relationship matrix between genotypes was considered in Equation 4. After estimation of effects and variance components, different Wald statistics were considered for significance test of α _*k*_ and (α *l*)_*kj*_. A general Wald statistic is defined as θ^'Var(θ^)−1θ^ , where $\hat{\theta }$ is a vector containing the estimates for the *p* fixed effects being tested and Var($Var{\left(\hat{\theta }\right)}^{-1}$ )^–1^ corresponds to the inverse of the variance–covariance matrix among tested effects estimators. The Var($Var(\hat{\theta })$ ) matrix is a submatrix obtained by extracting the *p* corresponding elements from the upper-left quadrant (**C**_11_) of the generalized inverse for the left-hand-side coefficient matrix in the mixed model equations (Henderson, 1975). For trait combinations with an unequal number of environments, **R** as well as the design matrices for fixed and random effects were modified as suggested by Henderson and Quaas (1976). Wald statistics follow a χ ^2^_*p*_ distribution. We performed a global Wald test for the α _*k*_ effects (*H*_0_: α _*k*1_=α _*k*2_=0), followed by an individual test for each α _*k*_ in the bivariate model. Markers were declared as potentially pleiotropic for two traits only when the α _*k*_ effects were significant at the global and at the two post-hoc individual tests. *R*^2^ values were computed by fitting all significant markers for each trait combination using a multiple-regression model with one of the traits as dependent variable. Markers with the lowest global Wald test *P*-values entered first in the model. Subsequently, *R*^2^ values were divided by the respective entry mean-based heritability; thus, they measure the proportion of genetic variance explained by a marker. This last procedure allowed direct comparisons among *R*^2^ values for different traits. A global Wald test for the (α *l*)_*kj*_ effects of both traits together and one test for each trait separately were performed to study marker×environment interactions. In addition to false discovery rate (FDR; Benjamini and Hochberg, 1995) and Bonferroni methods, we considered the effective number of independent markers (*M*_eff_) approach proposed by Gao *et al.* (2008) for genome-wide multiple-test correction. Basically, this method is similar to the Bonferroni approach, but the whole number of markers is replaced by the *M*_eff_ when correcting the nominal level of significance of tests. For this purpose, pairwise linkage disequilibrium (LD) matrices in the form of *r*^2^ were computed for each of the 21 wheat chromosomes using SNPs with unique genetic map positions (Wang *et al.*, 2014) along with functional markers. Later, principal component analysis was applied to each matrix and the number of eigenvalues needed to explain 95% of matrix variation was recorded. By adding these 21 values together, an *M*_eff_=3257 was obtained. Furthermore, because of the high collinearity between associated markers mapping in the same region, the effective number of bivariate associations was computed using the same principle as for *M*_eff_.

A complete genome scan using Equation 4 and performing Wald tests with more than two traits would imply a very high computational load. Therefore, in cases where markers with significant associations for more than one two-trait combination, hereafter called higher order GY-syndrome markers, were found, only this subset of markers was subsequently evaluated for more than two traits. Since genome-wide distributions of *P*-values for the Wald test were unknown in these cases, only *M*_eff_ and the Bonferroni correction methods were considered for higher order GY-syndrome markers.

A bivariate two-dimensional scan based on the method originally proposed by Jiang and Zeng (1995) for linkage studies was implemented in the context of GWAS. Briefly, this method contrasts *H*_0_: *p*(1)=*p*(2), stating that the QTLs for two different traits are located at the same *m* locus, with *H*_1_: *p*(1)≠*p*(2), in which these two QTLs have different positions, i.e. *m*_1_ and *m*_2_. In principle, a pleiotropic model and a linkage model are compared in terms of their likelihoods. Since BLUEs across environments were considered as phenotypic data for the bivariate two-dimensional scan, Equation 4 is reduced to:

$$y_{ki}=\mu_{k}+\alpha_{k}\times m_{i}+g_{ki}+e_{ki}$$ (5)

in the case of the pleiotropic model, whereas:

$$y_{ki}=\mu_{k}+\left(\text{2}–k\right)\alpha_{\text{1}}\times m_{\text{1}i}+\left(k–\text{1}\right)\alpha_{\text{2}}\times m_{\text{2}i}+g_{ki}+e_{ki}$$ (6)

corresponds to the linkage model, with *k*=1 or 2 for the first and second traits included in the model, respectively. Provided that *****Y*****=(*y*_ 11_, *y*_12 _, ..., *y*_1*_n_*_, *y*_21 _, *y*_22 _,..., *y*_2*_n_*_)^*T*^, the likelihood functions of pleiotropy and linkage models [i.e. *f*_0_(***Y***) and *f*_1_(***Y***)] are multivariate normal density functions with means $\left(\begin{array}{c} 1_{n}\mu_{1}+m\alpha_{1}\\ 1_{n}\mu_{2}+m\alpha_{2} \end{array}\right)$ and $\left(\begin{array}{c} 1_{n}\mu_{1}+m_{1}\alpha_{1}\\ 1_{n}\mu_{2}+m_{2}\alpha_{2} \end{array}\right)$ , respectively, where **1**_*n*_ is an *n*-size vector containing only ones, whereas ***m***, ***m***_1_, and ***m***_2_ denote *n*-size vectors of marker profiles. In both *f*_0_(***Y***) and *f*_1_(***Y***), the covariance matrix is **G**_**0**_⊗**A**+**R**_**0**_⊗**I**_*n*_. The LR test is denoted as $LR=-2\;\ln\left(\frac{L_{0}}{L_{1}}\right)$ , where *L*_1_ is the maximum of the likelihoods for a linkage model in the two-dimensional space (*H*_1_), and *L*_0_ is the maximum of the likelihoods on the diagonal of the two-dimensional space that corresponds to *H*_0_. Then, the test statistic under *H*_0_ follows a χ ^2^_1_ distribution for which a nominal significance level of 0.05 was applied (Jiang and Zeng, 1995). The two-dimensional space was defined by considering an *r*^2^ window with values >0.5 between the surrounding markers and each potential pleiotropic marker. Only markers with unique genetic map positions were taken into account for this purpose. Moreover, two traits were simulated under close linkage or pleiotropy scenarios to investigate the influence of minor allele frequencies (MAFs) along with balanced and unbalanced percentages of genetic variance explained by QTLs (QTL size) on the power and Type I error of close linkage detection in the GABI-WHEAT population. In balanced scenarios, QTL sizes were the same for both simulated traits, whereas QTL sizes were larger (or smaller) for one of the traits under the unbalanced scenario. The influence of *r*^2^ values between QTL positions was considered as an additional factor in linkage scenarios. Marker information (Supplementary Table S2) and some general statistics from phenotypic analyses of the GABI-WHEAT population were considered during simulations. Methods for simulations are described in detail in the Supplementary data. Linear mixed models were solved using the ASReml-R package (Butler *et al.*, 2009), and all computational methods were implemented within R environment (R Core Team, 2016).

## Results

### Grain yield was significantly associated with various yield-syndrome traits

On a plot basis, GY was moderately heritable (*h*^2^_Plot_=0.44), while most GY-syndrome traits, except EW, had higher heritabilities than GY (Table 1). A broad phenotypic variation was observed for all traits (Supplementary Table S3), and GY ranged from 7.4 Mg ha^−1^ to 11.1 Mg ha^−1^. Phenotypically, GY was positively correlated (*P*-value <0.0001) with GPE and EW but was negatively associated (*P*-value <0.0001) with PH and TW (Table 1). Almost all phenotypic correlations among GY-syndrome traits were significant (*P*-value <0.05), except between EW and the two traits PH and HD. At the genetic level, GY–PH and GY–TW correlations shifted towards zero (Table 1), whereas GY–TGW, GY–GPE, and GY–EW correlations were positive (*P*-value <0.05).

**Table 1.** Matrix of plot-based heritabilities (*h*^2^_Plot_, underlined diagonal values), genetic (*cor*_*g*_) and phenotypic (*cor*_*p*_) correlations (lower and upper triangle values, respectively) for grain yield (GY, Mg ha^−1^), plant height (PH, cm), heading date (HD, days since 1 January), thousand grain weight (TGW, g), test weight (TW, kg hl^−1^), grains per ear (GPE), and ear weight (EW, g) in the population of 358 European winter plus 15 spring wheat varieties (GABI-WHEAT population) phenotyped in up to eight environments

*cor*_*g*_/*h*^2^_Plot_/*cor*_*p*_	GY	PH	HD	TGW	TW	GPE	EW
GY	0.44	–0.24***	0.07	0.09	–0.37***	0.26***	0.21***
PH	0.08	0.87	0.16**	0.21***	0.64***	–0.18**	–0.03
HD	–0.05	0.00	0.85	–0.42***	-0.26***	0.37***	0.07
TGW	0.15*	0.29***	–0.45***	0.70	0.23***	–0.51***	0.22***
TW	0.00	0.41***	–0.42***	0.17**	0.71	–0.32***	–0.14**
GPE	0.29**	–0.08	0.36***	–0.60***	–0.18*	0.59	0.56***
EW	0.25**	0.16	0.00	0.42***	–0.09	0.63***	0.35

*Significantly different from zero with a *P*-value <0.05.

**Significantly different from zero with a *P*-value <0.01.

***Significantly different from zero with a *P*-value <0.0001.

### Bivariate genome-wide association mapping revealed several marker–trait–trait associations

**Fig. 1.**
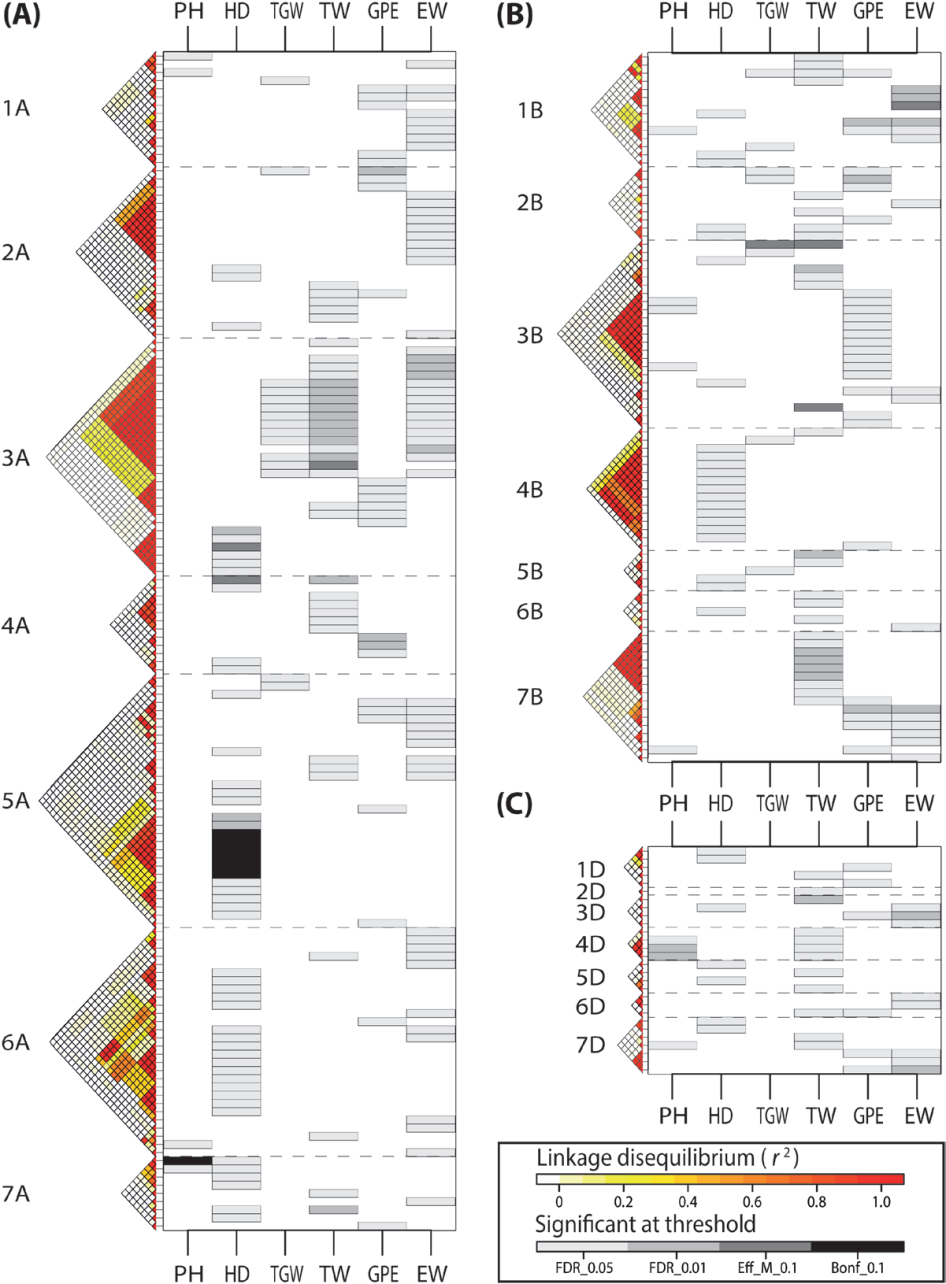
Genomic regions simultaneously associated with grain yield (GY) and at least one GY-syndrome trait as revealed by genome-wide association scans (GWAS) using bivariate models in the population of 358 European winter plus 15 spring wheat varieties (GABI-WHEAT population) phenotyped in up to eight environments and genotyped with 18 856 polymorphic markers. GY-syndrome traits corresponded to plant height (PH), heading date (HD), thousand grain weight (TGW), test weight (TW), grains per ear (GPE), and ear weight (EW). Significant associations of single nucleotide polymorphism (SNP) markers were positioned according to the reference genetic map of Wang *et al.* (2014), grouping them by genome: (A) A, (B) B, and (C) D. Functional markers for *Rht-B1b* (Ellis *et al.*, 2002) and *TaGW2-6B* (Qin *et al.*, 2014) were placed for convenience at the end of linkage groups 4B and 6B, respectively.

Among all available markers, a total of 345 had significant (FDR <0.05) bivariate associations with GY and at least one of the GY-syndrome traits (Fig. 1; Supplementary Tables S4–S9). With the exception of GY–GPE associations of SNP markers *Ku_c12191_1123*, *Tdurum_contig13646_225*, *wsnp_Ex_c22913_32130617*, and *wsnp_Ex_rep_c66274_64426834*, all the remaining bivariate associations presented significant marker×environment interactions for at least one trait. Marker–trait–trait associations were found on all wheat chromosomes (Fig. 1; Supplementary Fig. S1). Genome A concentrated a great proportion of them, with 144 markers presenting a total of 188 bivariate associations, whereas 106 and 37 associations were observed for B and D genomes, respectively, which were attributed to 87 and 28 markers for each genome, respectively. The minimum number of bivariate associations per chromosome was presented by chromosome 2D, with only one joint association for GY and TW, while 3A showed the maximum number of associations per chromosome, with up to 56 associations attributed to 29 markers. Depending on the trait pair, associated markers fitted in a multiple-regression model explained together 12.2–58.1% of GY phenotypic variation, while accounting for between 9.3% and 60.4% of GY-syndrome trait variation (Supplementary Table S10). Nonetheless, all distributions of single marker *R*^2^ values were L-shaped (Supplementary Fig. S2) and, in most cases, associated markers explained <1% of genetic variation for at least one of the traits (Supplementary Tables S4–S9).

**Fig. 2.**
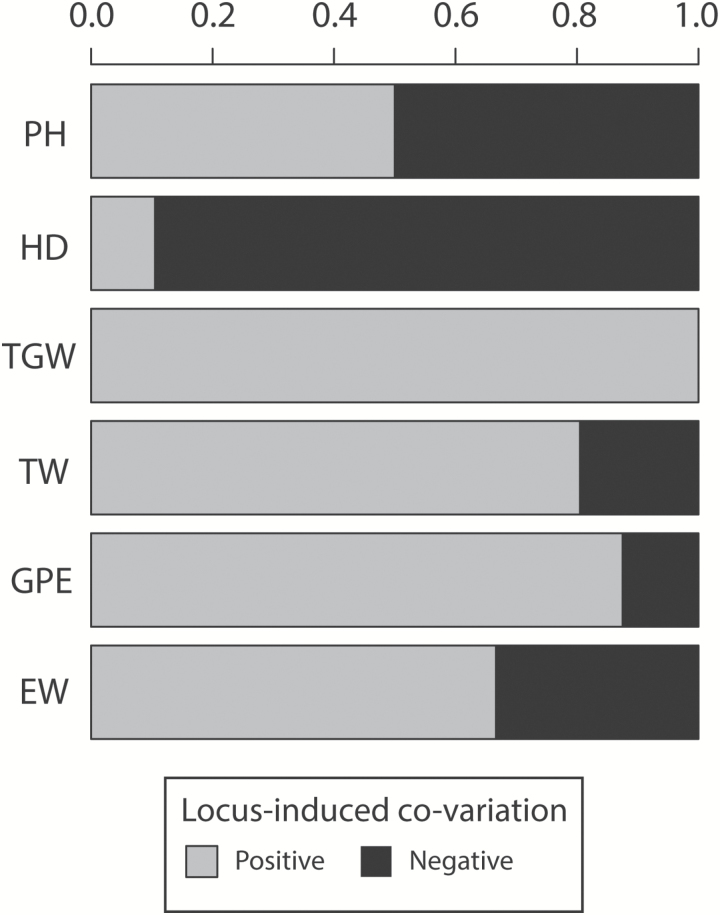
Sign distributions for locus-induced co-variation on traits (positive or negative) of the effective number of genetic factors simultaneously associated with grain yield (GY) and at least one GY-syndrome trait as revealed by genome-wide association scans (GWAS) using bivariate models in the population of 358 European winter plus 15 spring wheat varieties (GABI-WHEAT population). GY-syndrome traits corresponded to plant height (PH), heading date (HD), thousand grain weight (TGW), test weight (TW), grains per ear (GPE), and ear weight (EW).

A total of 18 significant SNP markers (FDR <0.05) were jointly associated with GY and PH (Supplementary Table S4). These markers represented a total of eight effective GY–PH associations (Supplementry Fig. S1) and half of them induced negative GY–PH co-variation (Fig. 2). Marker–trait–trait associations explained on average almost a three times larger proportion of genetic variation for PH than for GY (2.1% versus 0.8%), with the highest *R*^2^ for PH attributed to marker *RAC875_rep_c105718_304*. This marker accounted for 15.3% and 1.2% of PH and GY variation, respectively (Supplementary Table S4).

For GY and HD, 93 SNP markers were jointly associated with both traits at FDR <0.05 (Supplementary Table S5), which depicted an estimated total of 40 GY–HD effective associations (Supplementary Fig. S1). Most GY–HD associations (89% of effective associations) induced negative co-variation between these two traits (Fig. 2). Markers with GY–HD associations explained on average a comparable percentage of variation for GY and HD (0.9% of genetic variation in both cases). Nevertheless, effect sizes on each trait were often unbalanced at the individual marker level (Supplementary Table S5). For example, marker *wsnp_Ku_c16432_25320146* explained 9.2% of variation in GY but only 0.6% of HD variation.

Significant (FDR <0.05) bivariate associations for GY and TGW were observed for 28 SNP markers (Supplementary Table S6), representing an estimated total of 11 effective GY–TGW associations (Supplementary Fig. S1). All these bivariate associations induced positive co-variation between GY and TGW (Fig. 2). Interestingly, significant markers explained on average three times as much of the genetic variance for GY (1.4%) than for TGW (0.5%), with the highest GY *R*^2^ attributed to SNP *BS00028033_51*. This marker explained 4.6% of variation in GY but accounted for 0.1% of TGW variation (Supplementary Table S6).

Bivariate GWAS for GY and TW revealed 116 SNP markers simultaneously associated with these traits at FDR <0.05 (Supplementary Table S7). GY–TW-associated markers showed an estimated total of 40 effective associations (Supplementary Fig. S1), with the majority of them (80% of effective associations) inducing positive trait–trait co-variation (Fig. 2). Regarding *R*^2^ values, markers explained on average a comparable proportion of GY and TW variation (0.6% and 0.7% of genetic variation, respectively). Nonetheless, *R*^2^ values for GY and TW were again often asymmetric at the single SNP level (Supplementary Table S7).

GY–GPE associations were found to be significant (FDR <0.05) for 70 markers (Supplementary Table S8), which represented an estimated total of 34 effective bivariate associations (Supplementary Fig. S1). Most of these effective associations (88%) induced positive GY–GPE co-variation (Fig. 2). Although the percentages of variation explained by these markers were on average comparable between GY and GPE (1.1% and 0.8% of genetic variation, respectively), effect sizes on each trait were again often asymmetric at the single marker level (Supplementary Table S8). The GY–GPE association found on chromosome 4B corresponded to the *Rht-B1* locus (Ellis *et al.*, 2002). *Rht-B1* explained 0.3% and 0.1% of GY and GPE variation, respectively, and the dwarfing allele presented positive effects on both traits.

The number of markers with significant (FDR <0.05) GY–EW joint associations amounted to 114 (Supplementary Table S9), which showed an estimated total of 35 effective associations (Supplementary Fig. S1). More than a half of them (67% of effective associations) induced positive co-variation between GY and EW (Fig. 2). Similar to previous cases, even though average *R*^2^ values were comparable for GY and EW (0.7 and 0.8% of genetic variance, respectively), *R*^2^ values for each trait were often unequal at a single marker level (Supplementary Table S9). The unique GY–EW association found on chromosome 6B corresponded to a marker developed for the *TaGW2-6B* locus (Qin *et al.*, 2014). This particular marker explained 0.3% and 0.2% of GY and EW variation, respectively, with the *Hap-6B-1* haplotype having positive effects on both traits.

### Simulation study on the power to distinguish close linkage from pleiotropy

The power to distinguish close linkage from pleiotropy ranged in our simulation study from 3% to 58% and increased with larger QTL sizes and more intermediate allele frequencies, but was hampered by tight LD between QTL positions (Table 2). Similar trends were found when simulating two QTLs with asymmetric sizes (Supplementary Table S11). Some exceptions to these main effects, such as power increments observed when the LD increased from *r*^2^ ~0.55 to 0.70 at MAF ~0.13 with QTLs explaining 10% of genetic variation for both traits, could be attributed to interactions among these factors along with other variables ignored during simulations. Regarding Type I error; that is, wrongly rejecting pleiotropy, no clear trends related to the different levels of MAF and QTL sizes were observed (Supplementary Table S12). Across all tested conditions, the average Type I error was 0.07, with an standard deviation of 0.04.

**Table 2.** Power of the test developed by Jiang and Zeng (1995) to differentiate close linkage from pleiotropy in linkage-simulated scenarios considering different levels of minor allele frequency (MAF), balanced percentage of explained genetic variation (QTL size) for each of the two simulated traits, linkage disequilibrium (*r*^2^), and marker profiles of the 358 European winter plus 15 spring wheat varieties (GABI-WHEAT population). Each value corresponds to the proportion of times in which *H*_0_: *p*(1)=*p*(2) was rejected in 100 simulated replicates

MAF	QTL size	*r* ^2^		
		~0.55 (0.53–0.56)	~0.70 (0.68–0.72)	~0.91 (0.90–0.93)
~0.06 (0.06–0.07)	15	0.23	0.17	0.12
	10	0.17	0.09	0.07
	5	0.04	0.05	0.03
~0.13 (0.11–0.14)	15	0.37	0.36	0.09
	10	0.26	0.36	0.15
	5	0.12	0.15	0.09
~0.22 (0.20–0.24)	15	0.40	0.40	0.18
	10	0.36	0.31	0.13
	5	0.35	0.21	0.09
~0.46 (0.44–0.48)	15	0.58	0.41	0.31
	10	0.37	0.38	0.21
	5	0.23	0.20	0.11

### Two-dimensional scan to distinguish close linkage from pleiotropy

**Fig. 3.**
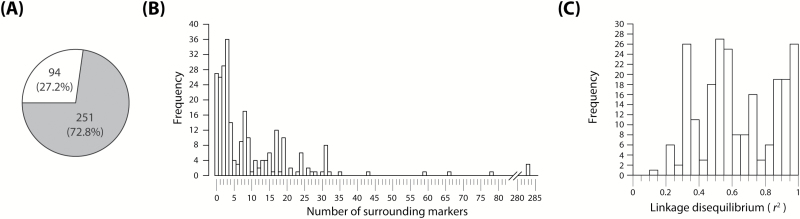
General statistics for markers considered during the two-dimensional scan to test close linkage versus pleiotropy in the population of 358 European winter plus 15 spring wheat varieties (GABI-WHEAT population). (A) Proportion of markers simultaneously associated with grain yield (GY) and at least one GY-syndrome trait having (shaded) or lacking unique genetic positions in wheat genomes according to past studies (Ellis *et al.*, 2002; Qin *et al.*, 2014; Wang *et al.*, 2014). (B) Frequency distribution of the number of surrounding markers in the vicinity (with linkage disequilibrium *r*^2^>0.5) of associated markers with unique genetic positions. (C) Distribution of frequencies for the minimum *r*^2^ value observed between markers within each vicinity window.

From the total of 345 markers with significant bivariate associations, a subsample of 251 markers had unique genetic map positions (Fig. 3A). A further 27 markers were discarded because of the lack of surrounding linked markers (*r*^2^>0.5). Thus, 224 markers were considered for the tests of close linkage versus pleiotropy (Fig. 3B; Supplementary Tables S4–S9). For these targeted markers, the number of surrounding linked markers ranged from 1 to 283, with an average of 13.6. According to the pairwise LD decay (Supplementary Fig. S3), surrounding markers are expected to map between 0 cM and 2 cM away from each targeted marker.

**Fig. 4.**
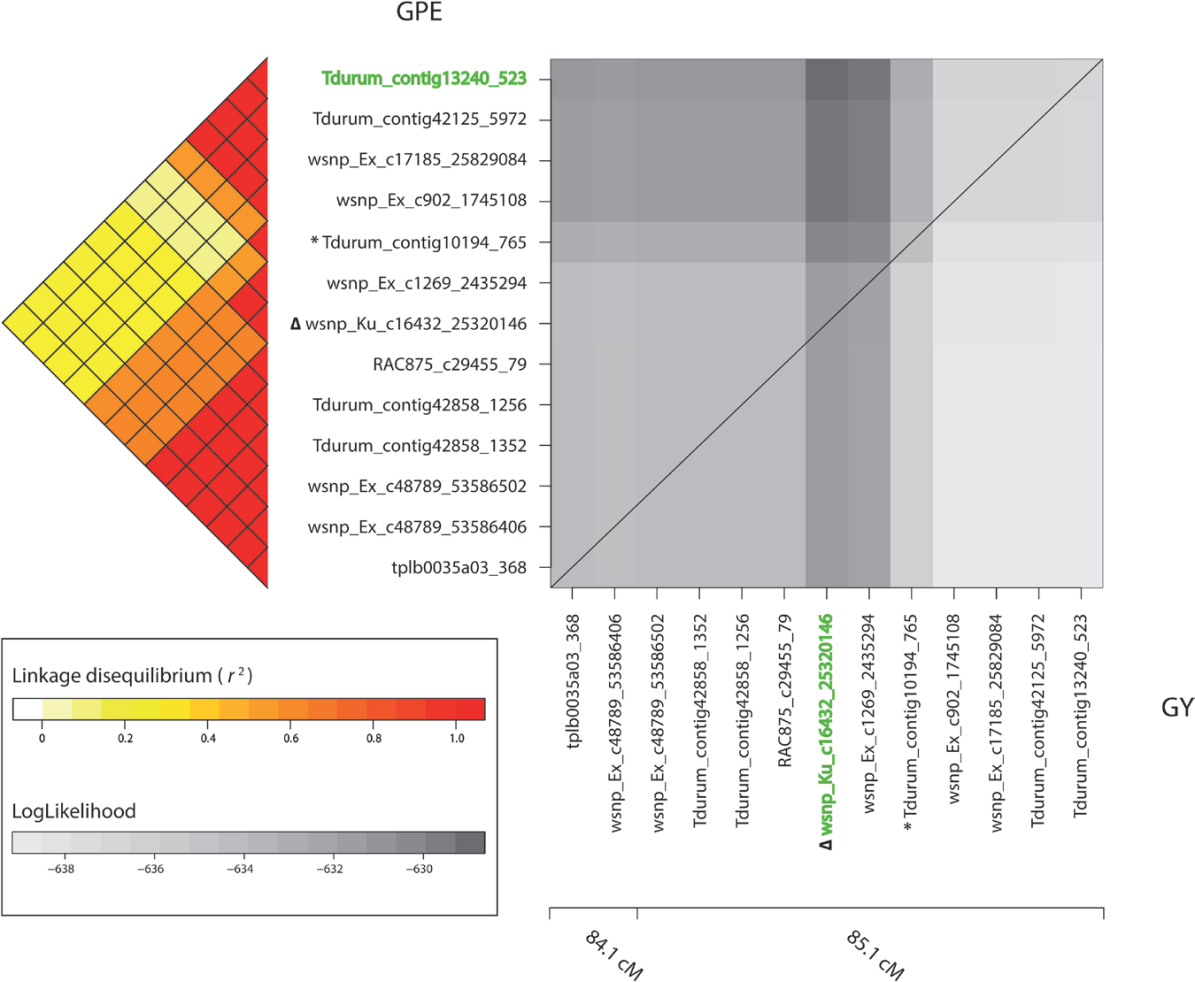
Landscape of bivariate likelihoods (log-likelihoods) during the two-dimensional scan to distinguish close linkage from pleiotropy in the vicinity of *Tdurum_contig10194_765* (indicated with an asterisk), a locus simultaneously associated with grain yield (GY) and grains per ear (GPE) on chromosome 6A (Fig. 1A; Supplementary Table S8). Likelihoods pertaining to pleiotropy models were maximized at marker *wsnp_Ku_c16432_25320146* (denoted with Δ), whereas likelihoods for linkage models were maximized at the combination of *wsnp_Ku_c16432_25320146* and *Tdurum_contig13240_523* (highlighted in green), with these last two markers carrying the effects on GY and GPE, respectively. The log-likelihood ratio test of Jiang and Zeng (1995) using maximized likelihoods rejected *H*_0_: *p*(1)=*p*(2) of pleiotropy at the nominal significance level of 0.05.

From all two-dimensional scans performed, we selected two extreme examples to illustrate the landscape of likelihoods for close linkage versus pleiotropy (Fig. 4; Supplementary Fig. S4). The two-dimensional scan for the GY–GPE-associated marker *Tdurum_contig10194_765* revealed that the maximum bivariate likelihood is clearly placed outside of the diagonal and, thus, the hypothesis of pleiotropy can be rejected (*P*-value <0.05). In contrast, for marker *IACX8108* jointly associated with GY and HD, the maximum bivariate likelihood was found on the diagonal; therefore, the hypothesis of pleiotropy cannot be rejected (Supplementary Fig. S4). Interestingly, the two-dimensional scan distinguished only three cases of close linkage from pleiotropy (Table 3). Besides the illustrative example of *Tdurum_contig10194_765*, the hypothesis of pleiotropy was rejected for *BS00003586_51* and *Tdurum_contig30930_184* corresponding to markers with significant GY–TGW and GY–EW bivariate associations, respectively.

**Table 3.** Marker–trait associations, chromosome location (Chr.), genetic positions (Pos., cM), number of surrounding markers (*N*), linkage disequilibrium (*r*^2^), and genetic distance (Dist., cM) pertaining to cases in which the log-likelihood ratio test of Jiang and Zeng (1995) rejected pleiotropy [*H*_0_: *p*(1)=*p*(2), *P*-value] in the population of 358 European winter plus 15 spring wheat varieties (GABI-WHEAT population)

Original association^*a*^					Disentangled close linkage								
Marker	Traits^*b*^	Chr.	Pos. (cM)^*c*^	*N* ^*d*^	*P*-value	Marker 1 (M1)	Pos. (cM)	Trait	Marker 2 (M2)	Pos. (cM)	Trait	*r* ^2^	Dist. (cM)^*e*^
*Tdurum_contig* *10194_765*	GY, GPE	6A	85.1	12	0.028	*wsnp_Ku_c16432_* *25320146*	85.1	GY	*Tdurum_contig* *13240_523*	85.1	GPE	0.12	0.0
*Tdurum_contig* *30930_184*	GY, EW	2B	108.0	2	0.049	*Tdurum_contig* *30930_184*	108.0	GY	*wsnp_JD_rep_* *c67103_42432235*	104.0	EW	0.58	4.0
*BS00003586_51*	GY, TGW	5B	144.1	2	0.021	*BS00098520_51*	144.1	GY	*BS00003586_51*	144.1	TGW	0.54	0.0

^*a*^Bivariate marker–trait associations as originally found by multiple-trait genome wide association mapping (MT-GWAS) in the GABI-WHEAT population (Fig. 1; Supplementary Tables S6, S8, S9).

^*b*^Traits involved in bivariate associations: grain yield (GY), thousand grain weight (TGW), grains per ear (GPE), and ear weight (EW).

^*c*^Genetic positions according to the reference map published by Wang *et al.* (2014).

^*d*^Number of markers in the vicinity of markers with bivariate associations (*r*^2^>0.5).

^*e*^Genetic distance as well as *r*^2^ values were calculated between M1 and M2.

### Higher order grain yield-syndrome markers

A total of 78 markers presented significant bivariate associations in more than one GY plus GY-syndrome trait combination (Fig. 1; Supplementary Tables S4–S9), suggesting that these markers are simultaneously influencing GY and two or more GY-syndrome traits. To elucidate this, marker effects were tested for their significance by sequentially adding more traits to an MT model according to bivariate results. First, three-trait models were considered; however, after multiple-test corrections, only one of the 78 markers remained significant for all traits in the model (data not shown). In detail, the SNP marker *IACX6214*, located at 33.7 cM on chromosome 3B, was significantly and simultaneously associated with GY, TGW, and TW (*M*_eff_-corrected *P*-value <0.1). This marker has negative minor allele effects on each trait, and could explain 0.02, 0.4, and 1.8% of genetic variation in GY, TGW, and TW, respectively, when included alone within a regression model for each trait. Nonetheless, the lack of surrounding linked markers (*r*^2^>0.5) for *IACX6214* precluded us from disentangling the pleiotropic nature of its simultaneous effects on GY, TGW, and TW.

## Discussion

### The power to distinguish linkage from pleiotropy is driven by minor allele frequencies, QTL size, and linkage disequilibrium

Across all simulated close linkage scenarios, it was observed that increasing MAF, along with QTL sizes and decreasing *r*^2^ levels between QTL positions, have a positive effect on the power to differentiate linkage from pleiotropy (Table 2; Supplementary Table S11). Our findings agree with past simulation studies considering various QTL sizes or different genetic distances/LD values between two QTLs (Lebreton *et al.*, 1998; Knott and Haley, 2000; Varona *et al.*, 2004; Da Costa E Silva *et al.*, 2012; David *et al.*, 2013). To the best of our knowledge, studies directly assessing the influence of MAF on the power and Type I error rate to differentiate close linkage from pleiotropy are lacking. Nevertheless, increments in MAF improve QTL detection power in GWAS (for a review, see Myles *et al.* 2009) and, from our results, this positive influence will also apply to the ability to distinguish pleiotropy from linkage.

In general, past simulation studies have shown that Type I error of close linkage versus pleiotropy testing is unaffected or, at most, only marginally influenced by balanced changes in QTL sizes (Lebreton *et al.*, 1998; Knott and Haley, 2000; Varona *et al.*, 2004; David *et al.*, 2013); an observation also found in our study (Supplementary Table S12). In addition, Lebreton *et al.* (1998) reported that unequal QTL sizes could increase Type I error by using a confidence interval approach to test pleiotropy versus linkage; however, this was not consistently observed by us. Nonetheless, since pleiotropy and linkage models of Jiang and Zeng (1995) are not exactly nested, asymptotical distributions of the LR test under *H*_0_ may deviate from χ^2^_1_; hence, alternative procedures have been suggested for significant threshold derivation in linkage studies (Almasy *et al.*, 1997; Williams *et al.*, 1999; Calinski *et al.*, 2000; Knott and Haley, 2000; Varona *et al.*, 2004; Da Costa E Silva *et al.*, 2012). Nevertheless, the average Type I error rate of 0.07 ± 0.04 observed during our simulations is comparable with the nominal level of 0.05 and also with the 0.1 value originally observed by Jiang and Zeng (1995), thus reflecting an acceptable test performance by considering standard significant thresholds, at least with our simulated scenarios.

### Grain yield is genetically correlated with thousand grain weight, grains per ear, and ear weight

Correlations at the phenotypic level would not necessarily resemble those at the genetic level, and vice versa. This is mainly because phenotypic correlations are not a direct function of genetic correlations, while being also dependent on the environmental correlation along with trait heritabilities (Falconer and Mackay, 1996). Such discrepancies were also observed in the present study (Table 1). For instance, while GY and PH were negatively correlated at the phenotypic level, they were not genetically correlated. Positive effects of reduced PH by improving harvest index and lodging resistance of high-yielding semi-dwarf wheat plants adapted to intensive agricultural practices have been long recognized (Borlaug, 1968; Austin *et al.*, 1980; Börner *et al.*, 1993; Brancourt-Hulmel *et al.*, 2003; Kuchel *et al.*, 2007; Casebow *et al.*, 2016; Kowalski *et al.*, 2016). The null genetic correlation between GY and PH can be attributed to the fact that varieties in the GABI-WHEAT population were already adapted by wheat breeders to perform in European agroecosystems. Moreover, since HD plays an important role in wheat adaptation as a crop (Worland, 1996), breeding efforts for adaptation can also explain the null correlation between HD and GY (Table 1). In relation to TW, this trait was negatively correlated with GY at the phenotypic level, while the genetic correlation was not significant between these traits. TW is a trait related to milling yield because it gives an indication of the soundness of wheat grains (Matsuo and Dexter, 1980). Nonetheless, its association with GY is still controversial (Huang *et al.*, 2006; Rattey *et al.*, 2009; Kamran *et al.*, 2014; Bordes *et al.*, 2014). Interestingly, despite the fact that GY–PH, GY–HD, and GY–TW genetic correlations were practically zero (Table 1), we could find pleiotropic associations for these trait pairs (Fig. 2). Since pleiotropic QTLs can induce positive/negative genetic co-variance among two traits if QTL effects on these traits have the same/opposite sign, these observed null genetic correlations could be simply the net effect of loci whose effects cancelled each other out. Regarding significant genetic correlations among GY and traits TGW, GPE, and EW, their positive associations confirm their main roles as GY components (e.g. Huang *et al.*, 2006; Kuchel *et al.*, 2007; Kumar *et al.*, 2007; Cuthbert *et al.*, 2008; Rattey *et al.*, 2009; Neumann *et al.*, 2011; Bennett *et al.*, 2012; Simmonds *et al.*, 2014; Sukumaran *et al.*, 2015).

### The pleiotropic architecture of wheat yield-syndrome was dissected more as a function of pleiotropy rather than close linkage

Past linkage studies on crop plants have shown that disentangling linkage from pleiotropy is quite difficult and sometimes even remains unsolved (Lebreton *et al.*, 1998; Calinski et al., 2000; Balestre *et al.*, 2012). In particular, Calinski *et al.* (2000) and Balestre *et al.* (2012) observed that although there was some evidence favoring linkage, this was not enough to discard pleiotropy completely as the underlying mechanism for maize GY-syndrome. Similarly, among all bivariate associations found in our study (Supplementary Tables S4–S9; Supplementary Fig. S1), only three of them were due to close linkage (Table 3). Nonetheless, we presume that the importance of pleiotropy could be overestimated, at least for the traits considered in this study. First, the majority of associated markers explained <1% of trait variation (Supplementary Tables S4–S9; Supplementary Fig. S2), which, as previously discussed, is expected to lead to low power to differentiate linkage from pleiotropy. Secondly, the distribution of *r*^2^ values between the most distant markers within each vicinity window showed in general values >0.5 (Fig. 3C), thus jeopardizing the fitting of linkage models using pairs of more distant markers. Although relaxing the LD window for the inclusion of less linked markers would be a simple solution, this would increase the risk of falsely declaring linkage in the presence of true pleiotropy, because one main prerequisite underlying our methodology is that each bivariate-associated marker is simultaneously detecting signals of two QTLs in the surroundings. Alternatively, a more reliable solution would be to increase marker density and population size, since these two factors may have limited the ability to differentiate close linkage from pleiotropy in the current study (Lebreton *et al.*, 1998; Varona *et al.*, 2004; David *et al.*, 2013).

### Rht-B1 *and* TaGW2-6B *showed pleiotropic effects on yield-syndrome*

From the 24 assessed functional markers (Supplementary Table S1), *Rht-B1* and *TaGW2-6B* were significantly associated with GY-syndrome. One and 66 surrounding linked markers (*r*^2^>0.5) were found for *Rht-B1* and *TaGW2-6B*, respectively (Supplementary Tables S8, S9). Within each vicinity window, the LR test could not reject the hypothesis of pleiotropy for these loci. Therefore, we conclude that the pipeline of MT analyses used in the present study confirmed the pleiotropic nature of *Rht-B1* and *TaGW2-6B* effects on GY-syndrome of wheat. The pleiotropic roles of these two loci are discussed in the following paragraphs.

Dwarfing loci *Rht8*, *Rht-B1*, and *Rht-D1* have been extensively associated with variation in GY, along with other GY-syndrome traits (Austin *et al.*, 1980; Börner *et al.*, 1993; Miralles and Slafer, 1995; Korzun *et al.*, 1998; Ellis *et al.*, 2002; Brancourt-Hulmel *et al.*, 2003; Kuchel *et al.*, 2007; Zanke *et al.*, 2014*b*, 2015; Casebow *et al.*, 2016; Kowalski *et al.*, 2016; Mohler *et al.*, 2016). Functional markers linked to these genes are available (Korzun *et al.*, 1998; Ellis *et al.*, 2002) and were used to characterize the GABI-WHEAT population in past works (Kollers *et al.*, 2013*a*, *b*). A bivariate association was found for *Rht-B1*, with the dwarfing allele (*Rht-B1b*) simultaneously increasing GY and GPE (Supplementary Table S8). As jointly concluded from past studies, it seems that although genotypes carrying *Rht-B1b* can produce more GPE, its positive association with GY would highly depend on effects of *Rht-B1b* on other GY-syndrome traits, along with counterbalancing effects of higher GPE values on grain weight *per se* and *Rht-B1b* interactions with environments (Börner *et al.*, 1993; Kuchel *et al.*, 2007; Casebow *et al.*, 2016). Apparently, increases in GPE produced by *Rht-B1b* in the GABI-WHEAT population were strong enough to compensate for any negative feedback on GY, resulting in a positive net effect on these two traits.

Based on 11 SNPs, Qin *et al.* (2014) described four different haplotypes, *Hap-6B-1* to *Hap-6B-4*, for locus *TaGW2-6B*. In their study *Hap-6B-1* was associated with higher average TGW, this superiority being attributed to increased grain length, width, and/or thickness. The GABI-WHEAT population was characterized in Zanke *et al.* (2015) using a marker designed by Qin *et al.* (2014) capable of distinguishing *Hap-6B-1* from the other three haplotypes. As in the present study, Zanke *et al.* (2015) found no association of *TaGW2-6B* with TGW. Recently, Mohler *et al.* (2016) found a minor TGW QTL at *TaGW2-6B* in a biparental doubled haploid population, but *Hap-6B-2* presented a higher TGW compared with *Hap-6B-1*. Moreover, in the current study, *Hap-6B-1* simultaneously increased GY and EW (Supplementary Table S9). Future studies should clarify the mechanisms that allowed *TaGW2-6B* to influence GY and EW without causing significant changes in TGW in the GABI-WHEAT population. Nevertheless, although a GY component different from TGW was involved, our findings confirm the positive role of *Hap-6B-1* on GY-syndrome.

### Pleiotropic effects are more environmentally stable for plant height and heading date than for grain yield

In past studies, GY QTLs have been often reported as environmentally unstable (e.g. Kuchel *et al.*, 2007; Kumar *et al.*, 2007; Cuthbert *et al.*, 2008; Reif *et al.*, 2011; Bennett *et al.*, 2012; Zhang *et al.*, 2014). In parallel, Kowalski *et al.* (2016) showed that while pleiotropic effects of *Rht8* are highly stable on PH, this was not the case on GY. In contrast, Simmonds *et al.* (2014) found two co-located QTLs on chromosome 6A influencing GY and TGW, respectively, which presented different environmental instability patterns for each trait. We further compared the environmental stability of marker pleiotropic effects on traits measured in the same number of environments (i.e. GY, PH, and HD). For this, we fitted a univariate multiple regression model including main environmental and marker effects plus their interactions considering the same data used for MT-GWAS. For GY–PH- and GY–HD-associated markers, the proportion of GY variation explained by main marker effects together corresponded to 1.6 and 0.6 times that attributed to interactions, respectively, while these ratios were 29.4 and 9 for PH and HD, respectively. This clearly indicates that marker×environment interactions played a more important role on GY than on PH or HD, and points to an increased environmental stability of pleiotropic effects on PH or HD than on GY.

### Disentangling close linkage from pleiotropy underlying yield-syndrome: implications in applied multiple-trait breeding

Markers *Tdurum_contig10194_765*, *BS00003586_51*, and *Tdurum_contig30930_184*, presenting bivariate GY–GPE, GY–TGW, and GY–EW associations, respectively, were disentangled as cases of close linkage (Table 3). GY and GPE QTLs of *Tdurum_contig10194_765* were displaced to markers having an *r*^2^ of 0.12, whereas repositioning of GY and EW QTLs of *BS00003586_51* and *Tdurum_contig30930_184*, respectively, resulted in close linkage cases of markers having *r*^2^ values of 0.54 and 0.58, respectively. We further investigated the frequencies of the four potential haplotypes generated by combining different alleles at these loci (Supplementary Fig. S5). For GY–GPE and GY–TGW disentangled cases, haplotypes maximizing both GY along with GPE or TGW were the most frequent, representing 76.5% and 73.8% of the GABI-WHEAT population, respectively, and suggesting that these haplotypes have been positively selected by wheat breeders. In contrast, the most frequent haplotype (52.6% in the GABI-WHEAT population) for the GY–EW disentangled case decreased both traits simultaneously; thus, efforts should be allocated to reverse this situation in MT improvement of these traits. In addition, blindly performing MT-MAS using markers *BS00003586_51* and *Tdurum_contig30930_184* would allow the selection of undesired haplotypes, corresponding to 11.6% and 7.7% of the selected fraction, respectively. Although these last magnitudes are relatively small, they illustrate the potential problems caused by blindly using spurious pleiotropic associations and the importance of disentangling close linkage from pleiotropy in MT-MAS. Moreover, most bivariate associations remained as pleiotropic in our study (Supplementary Tables S4–S9) and, provided they are truly pleiotropic, MT-MAS using markers whose trait induced co-variation (Fig. 2) agrees with breeding goals should be straight forward. Nonetheless, we also found bivariate associations for which this was not the case. For instance, 33% of GY–EW effective associations induced negative trait co-variations. For these cases, using additional markers that complement or mitigate undesired effect(s) of pleiotropic markers could be a solution (Chen and Lübberstedt, 2010). Modeling pleiotropic epistasis as done recently in humans (Zhang *et al.*, 2016) could bring a better understanding of how this complementarity works.

### Conclusion

We assessed the GY-syndrome of bread wheat through MT-GWAS approaches in a population of 372 varieties adapted to European environments and genotyped with 18 832 SNPs plus 24 polymorphic functional markers. We conclude that distinguishing pleiotropy from close linkage underlying the pleiotropic architecture of GY-syndrome is very challenging, especially because of the small sizes of QTLs influencing this complex trait. Our results should be considered as a starting point for further complementary biological validations.

## Supplementary data

Supplementary data are available at *JXB* online. 

Simulation methods. Details for simulations on power and Type I error of close linkage detection.

Table S1. Details of functional molecular markers used to characterize the GABI-WHEAT population.

Table S2. Details of markers used for pleiotropy and close linkage simulations.

Table S3. General statistics for BLUEs across environments.

Table S4. Details for markers with significant GY–PH associations and test of pleiotropy versus close linkage.

Table S5. Details for markers with significant GY–HD associations and test of pleiotropy versus close linkage.

Table S6. Details for markers with significant GY–TGW associations and test of pleiotropy versus close linkage.

Table S7. Details for markers with significant GY–TW associations and test of pleiotropy versus close linkage.

Table S8. Details for markers with significant GY–GPE associations and test of pleiotropy versus close linkage.

Table S9. Details for markers with significant GY–EW associations and test of pleiotropy versus close linkage.

Table S10. Percentage of phenotypic and genetic variation explained in total by associated markers

Table S11. Power to differentiate close linkage from pleiotropy in the case of unequal QTL sizes for two simulated traits.

Table S12. Type I error of pleiotropy rejection.

Fig. S1. Number of effective bivariate associations.

Fig. S2. Frequency distributions of *R*^2^ values.

Fig. S3. Decay of intrachromosomal pairwise *r*^2^.

Fig. S4. Two-dimensional scan in the surroundings of marker *IACX8108*.

Fig. S5. Haplotype frequencies for cases disentangled as close linkage.

## Supplementary Material

Supplementary_Tables_S1_S3_S10_S12_Figures_S1_S5

Supplementary_Tables_S4_S9

## Abbreviations:

BLUE: best linear unbiased estimator
EW: ear weight
FDR: false discovery rate multiple-test correction
GPE: grains per ear
GWAS: genome-wide association study
GY: grain yield
HD: heading date
LD: linkage disequilibrium
LR: likelihood ratio
MAF: minor allele frequency
MAS: marker-assisted selection
MT: multiple-trait
PH: plant height
QTL: quantitative trait locus
SNP: single nucleotide polymorphism
ST: single-trait
TGW: thousand grain weight
TW: test weight.
