# Supplementary material for: The roles of pleiotropy and close linkage as revealed by association mapping of yield and correlated traits of wheat (Triticum aestivum L.)
Source: J Exp Bot. 2017 Jul 21;68(15):4089–101. doi: 10.1093/jxb/erx214 (PMC5853857; doi:10.1093/jxb/erx214)
Supplement: Supplementary_Tables_S1_S3_S10_S12_Figures_S1_S5 [file erx214_suppl_supplementary_tables_s1_s3_s10_s12_figures_s1_s5.pdf]

Supplementary material

This document contains:

Methods for simulations explained in detail

Supplementary Tables S1-S3 and S10-S12

Supplementary Figures S1-S5

For Supplementary tables S4-S9, please refer to file “Supplementary_Tables_S4_to_S9.xlsx” also uploaded among the supplementary material

Simulation methods

Firstly, the following parameters were estimated from the phenotypic analyses of traits grain yield and ear weight in the GABI-WHEAT population: μ_k: population mean for the kth trait; h_k^2: entry-mean based heritability of the kth trait; $\sigma_{g_k}^2$: genetic variation of the kth trait; ρ_g: genetic correlation between the two traits; $\sigma_{e_k}^2 = \frac{1-h_k^2}{h_k^2}\sigma_{g_k}^2$: residual variance of the kth trait; and ρ_e: residual correlation between the two traits. Denoting each element of $\mathbf{R_0}$ in Eq. (3) (please see the Phenotypic analyses section included in Materials and Methods) as $r_{kk'}$; ρ_e was estimated as $\frac{r_{12}}{\sqrt{r_{11} \times r_{22}}}$.

During the sampling of quantitative trait loci (QTL) effects, we assumed p_k values, which corresponded to the proportion of genetic variance explained by a QTL for the kth trait. In scenarios of balanced QTL sizes, both p_k values were 0.05, 0.10 or 0.15, whereas in scenarios of unbalanced QTL sizes, p_k was fixed to 0.15 for one trait and was decreased to 0.10 and 0.05 for the second trait. For the sampling of QTL effects, the following **G** matrix was considered:

$$\mathbf{G} = \begin{bmatrix} p_1 * \sigma_{g_1}^2 & \rho_g * \sigma_{g_1} * \sigma_{g_2} * \sqrt{p_1 * p_2} \\ \rho_g * \sigma_{g_1} * \sigma_{g_2} * \sqrt{p_1 * p_2} & p_2 * \sigma_{g_2}^2 \end{bmatrix}.$$

By means of single value decomposition (SVD) of **G** in the form: $\mathbf{G} = \mathbf{UDU'}$, where **U** corresponds to a matrix of eigenvectors and **D** is a diagonal matrix containing eigenvalues in the way $\mathbf{D} = \begin{bmatrix} d_1 & 0 \\ 0 & d_2 \end{bmatrix}$ (Searle, 2006), canonical variables x_k were subsequently sampled from a normal distribution in the way: $\boldsymbol{x} = \begin{bmatrix} x_1 \\ x_2 \end{bmatrix} \sim N(\mathbf{0}, \mathbf{D})$. Then, QTL effects ($\boldsymbol{z}$) were obtained by the operation $\mathbf{U}\boldsymbol{x} = \boldsymbol{z} = \begin{bmatrix} z_1 \\ z_2 \end{bmatrix}$.

The sampling of genetic background effects, i.e. the proportion of genetic variance not explained by QTL, was performed considering the following $\widetilde{\boldsymbol{A}}$ matrix:

$$\widetilde{\boldsymbol{A}} = \begin{bmatrix} (1-p_1) * \sigma_{g_1}^2 & \rho_g * \sigma_{g_1} * \sigma_{g_2} * \sqrt{(1-p_1) * (1-p_2)} \\ \rho_g * \sigma_{g_1} * \sigma_{g_2} * \sqrt{(1-p_1) * (1-p_2)} & (1-p_2) * \sigma_{g_2}^2 \end{bmatrix} \otimes \mathbf{A},$$

where $\otimes$ denotes the Kronecker product operator between matrices (Searle, 2006), **A** is the kinship matrix between genotypes estimated as $2 \times (\mathbf{J} - \mathbf{RD})$, with **J** denoting an $n \times n$ matrix whose every element is 1 and **RD** corresponding to the Rogers' distance matrix (Rogers, 1972) calculated from SNP profiles of n genotypes. In the present study, n corresponded to 372, which is the size of the GABI-WHEAT population. By means of SVD of $\widetilde{\boldsymbol{A}}$ in the way $\widetilde{\boldsymbol{A}} = \mathbf{W\Lambda W'}$, where $\mathbf{\Lambda} = \begin{bmatrix} \lambda_1 & \cdots & 0 \\ \vdots & \ddots & \vdots \\ 0 & \cdots & \lambda_{2n} \end{bmatrix}$, canonical variables $\boldsymbol{f}$ were sampled from a normal distribution as: $\boldsymbol{f} = \begin{bmatrix} f_1 \\ \vdots \\ f_{2n} \end{bmatrix} \sim N(\mathbf{0}, \mathbf{\Lambda})$. Later, background effects ($\boldsymbol{g}$) were obtained by the matrix product $\mathbf{W}\boldsymbol{f} = \boldsymbol{g} = \begin{bmatrix} g_1 \\ \vdots \\ g_{2n} \end{bmatrix}$.

The following **R** matrix was considered during the sampling of residual effects:

$$\mathbf{R} = \begin{bmatrix} \sigma_{e_1}^2 & \rho_e * \sigma_{e_1} * \sigma_{e_2} \\ \rho_e * \sigma_{e_1} * \sigma_{e_2} & \sigma_{e_2}^2 \end{bmatrix}.$$

Subsequently, by means of SVD of $\mathbf{R}$ in the form $\mathbf{R} = \mathbf{VEV}'$, with $\mathbf{E} = \begin{bmatrix} \ell_1 & 0 \\ 0 & \ell_2 \end{bmatrix}$, canonical variables $\boldsymbol{\varepsilon}_i$ were sampled in the way $\boldsymbol{\varepsilon}_i = \begin{bmatrix} \varepsilon_{i1} \\ \varepsilon_{i2} \end{bmatrix} \sim N(\mathbf{0}, \mathbf{E})$, with $i \in \{1, 2, \cdots n\}$. After this, residual effects ($\boldsymbol{e}_i$) were calculated as $\mathbf{V}\boldsymbol{\varepsilon}_i = \boldsymbol{e}_i = \begin{bmatrix} e_{i1} \\ e_{i2} \end{bmatrix}$.

Close linkage and pleiotropy scenarios were assessed during the simulation by assigning the two simulated QTL to two different but closely linked markers ($m_1$and m_2, respectively) in the case of linkage or by placing two QTL at the same marker position (m) in the case of pleiotropy. Then, the n phenotypic values ($\boldsymbol{Y}_k$) for the kth trait under close linkage simulations were computed as follows:

$$\boldsymbol{Y}_k = \begin{bmatrix} y_{k1} \\ \vdots \\ y_{kn} \end{bmatrix} = \mathbf{1}_n \mu_k + m_k z_k + \begin{bmatrix} g_{(k-1)n+1} \\ \vdots \\ g_{kn} \end{bmatrix} + \begin{bmatrix} e_{1k} \\ \vdots \\ e_{nk} \end{bmatrix},$$

where $\mathbf{1}_n$ is a vector of length n that contains only ones (Searle, 2006), m_k corresponds to an n-length vector of marker profiles to which the z_k effect was assigned and $k \in \{1, 2\}$. In pleiotropy simulations the phenotypic values for the kth trait were calculated as follows:

$$\boldsymbol{Y}_k = \begin{bmatrix} y_{k1} \\ \vdots \\ y_{kn} \end{bmatrix} = \mathbf{1}_n \mu_k + m z_k + \begin{bmatrix} g_{(k-1)n+1} \\ \vdots \\ g_{kn} \end{bmatrix} + \begin{bmatrix} e_{1k} \\ \vdots \\ e_{nk} \end{bmatrix}.$$

Markers $m_1$and m_2 were selected within the vicinity (with linkage disequilibrium, r^2 values > 0.5) of each m marker. During the selection of markers for the pleiotropy scenario we considered 0.07, 0.13, 0.24, and 0.45 minor allele frequency (MAF) levels, and also procured that these markers presented a similar number of surrounding markers, ranging from 19 to 26 markers in the vicinity. This approach facilitated the definition of four MAF regions in which we could subsequently select marker pairs according to three different r^2 levels (~0.55, 0.70 and 0.91) for the close linkage scenarios. Further details of markers selected for simulations can be found in Table S2. The combination of MAF, r^2 and $p_k$levels configured a total of 28 and 84 different genetic scenarios for the pleiotropy and close linkage simulations, respectively. By repeated sampling, each genetic scenario was replicated 100 times. For simplicity, it was assumed that a previous multiple-trait genome wide association study detected pleiotropic associations for each replication within each genetic scenario, and then, we performed a two-dimensional scan to test close linkage vs pleiotropy according to Jiang and Zeng (1995). The power to differentiate close linkage from pleiotropy was calculated as the number of times divided by 100 in which the hypothesis of pleiotropy (H_0) was rejected using close linkage simulations. In parallel, the Type I error rate was computed as the number of times divided by 100 that H_0 was rejected using pleiotropy simulations.

Table S1. Chromosome location, number of alleles, trait association and literature references of functional molecular markers used to characterize the population of 358 European winter plus 15 spring wheat varieties (GABI-WHEAT population).

Marker, locus or allele	Chromosome	Alleles	GABI-WHEAT[a]	Candidate for	Original reference
UMN19	1A	Biallelic	This study	Flour quality	Liu *et al.* (2008)
TaTGW6-A1	3A	Biallelic	This study	Thousand grain weight and yield	Hanif *et al.* (2016)
Xgwm369	3A	Multiallelic	Kollers *et al.* 2013*a*	Resistance to *Septoria tritici* blotch	Chartrain *et al.* (2005)
TaCWI-4A	4A	Biallelic	Zanke *et al.* 2015	Thousand grain weight and grains per ear	Jiang *et al.* (2015)
TaGW2-6A	6A	Biallelic	Zanke *et al.* 2015	Grain width and thousand grain weight	Su *et al.* (2011)
*TaSus1-7A*_CAPS_1185	7A	Biallelic	Zanke *et al.* 2015	Thousand grain weight	Hou *et al.* (2014)
*TaSus1-7A*_CAPS_3544	7A	Biallelic	Zanke *et al.* 2015	Thousand grain weight	Hou *et al.* (2014)
Glu-B1-1d	1B	Biallelic	This study	Flour quality	Schwarz *et al.* (2004)
Glu-B1-Bx7	1B	Biallelic	This study	Dough strength	Butow *et al.* (2004)
Viviparous-1B	3B	Multiallelic	This study	Pre-harvest sprouting	Xia *et al.* (2008)
Rht-B1b	4B	Biallelic	Kollers *et al.* 2013*b*	Plant height	Ellis *et al.* (2002)
TaGW2-6B	6B	Biallelic	Zanke *et al.* 2015	Thousand grain weight	Qin *et al.* (2014)
TaSus1-7B	7B	Biallelic	Zanke *et al.* 2015	Thousand grain weight	Hou *et al.* (2014)
UMN25	1D	Biallelic	This study	Flour quality	Liu *et al.* (2008)
UMN26	1D	Biallelic	This study	Flour quality	Liu *et al.* (2008)
Ppd-D1a	2D	Biallelic	Kollers *et al.* 2013*b*	Sensitivity to day length	Beales *et al.* (2007)
WMS261	2D	Multiallelic	Kollers *et al.* 2013*a*	Plant height	Korzun *et al.* (1998)
Rht-D1b	4D	Biallelic	Kollers *et al.* 2013*b*	Plant height	Ellis *et al.* (2002)
Pinb-D1	5D	Multiallelic	This study	Grain hardness	Huang and Röder (2005)
Puroindoline a	5D	Biallelic	This study	Grain hardness	Gautier *et al.* (1994)
Xgwm469-5D	5D	Multiallelic	Kollers *et al.* 2013*a*	Resistance to soil borne cereal mosaic virus	Perovic *et al.* (2009)
Ep-D1b	7D	Biallelic	This study	Resistance to eyespot	McMillin *et al.* (1986)[b]
TaGS-D1	7D	Multiallelic	Zanke *et al.* 2015	Thousand grain weight	Zhang *et al.* (2014)
XustSSR2001–7DL	7D	Biallelic	This study	Resistance to eyespot	Groenewald *et al.* (2003)

[a] Publication in which the GABI-WHEAT population was characterized using a particular marker
[b] Protocol available online at http://maswheat.ucdavis.edu/protocols/Eyespot/index.htm

Table S2. Number of markers in the vicinity (*N*), chromosome location (Chr.), genetic position (cM), minor allele frequency (MAF), linkage disequilibrium (r^2) and genetic distance (cM) of markers carrying two simulated quantitative trait loci (QTL) under pleiotropy and close linkage simulation scenarios.

Simulated scenario										
Pleiotropy					Close linkage					
Marker	*N*	MAF	Chr.	Position (cM)[a]	Marker 1 (M1)	MAF	Marker 2 (M2)	MAF	r^2	Distance (cM)[b]
Excalibur_c32170_145	19	0.07	3B	62.6	*wsnp_CD897414B_Ta_2_1*	0.06	*CAP12_rep_c4638_80*	0.06	0.56	0.3
					Kukri_c11905_89	0.07	*Excalibur_c3103_878*	0.06	0.70	0.0
					Excalibur_c3103_878	0.06	*RAC875_c25375_236*	0.06	0.90	0.3
wsnp_Ex_c48789_53586406	24	0.13	6A	84.1	*wsnp_JD_rep_c62949_40140212*	0.11	*RAC875_c29455_79*	0.14	0.53	1.0
					BS00064548_51	0.14	*RAC875_c691_1486*	0.13	0.72	2.6
					BS00065633_51	0.12	*wsnp_JD_rep_c62949_40140212*	0.11	0.90	1.3
RAC875_c15807_669	26	0.24	4B	69.8	*RAC875_c104414_76*	0.23	*Excalibur_c27948_1073*	0.20	0.55	1.5
					tplb0034b12_591	0.24	*RAC875_rep_c109069_89*	0.20	0.68	0.0
					Kukri_c32064_629	0.24	*Kukri_c15910_159*	0.23	0.93	0.0
Ku_c69999_111	23	0.45	6A	85.1	*Excalibur_c11578_324*	0.45	*Ku_c69999_111*	0.45	0.56	0.0
					Ku_c69999_111	0.45	*wsnp_Ex_c9502_15748469*	0.48	0.71	0.0
					BS00036397_51	0.44	*wsnp_Ex_c9502_15748251*	0.47	0.90	0.0

[a] Genetic positions (cM) obtained from Wang *et al.* (2014)

[b] Genetic distance as well as r^2 values were calculated between M1 and M2 of close linkage simulations

Table S3. Entry-mean based heritabilities (h^2_{Entry}) along with minimum (Min), mean, and maximum (Max) values of the best linear unbiased estimators (BLUEs) across environments for the population of 358 European winter plus 15 spring wheat varieties (GABI-WHEAT population) phenotyped in up to eight environments according to traits grain yield (GY, Mg ha^{-1}), plant height (PH, cm), heading date (HD, days since 1st January), thousand grains weight (TGW, g), test weight (TW, kg hL^{-1}), grains per ear (GPE), and ear weight (EW, g).

	General statistics			
Trait	h^2_{Entry}	Min	Mean	Max
GY	0.89	7.40	9.67	11.07
PH	0.99	69.5	87.2	110.7
HD	0.98	142.1	151.1	159.2
TGW	0.96	35.7	45.8	59.2
TW	0.96	71.8	79.2	84.5
GPE	0.82	33.5	50.2	66.5
EW	0.65	24.4	32.5	42.1

Table S10. Percentage of total explained (R^2_{Total}) phenotypic and genetic variation of markers being simultaneously associated (false discovery rate, FDR < 0.05) to grain yield (GY) and one GY-syndrome trait (Trait pair) in the population of 358 European winter plus 15 spring wheat varieties (GABI-WHEAT population). GY-syndrome traits corresponded to plant height (PH), heading date (HD), thousand grains weight (TGW), test weight (TW), grains per ear (GPE) and ear weight (EW).

		R^2_{Total} (%)[a]			
		Phenotypic		Genetic	
Trait pair	*N*	T1	T2	T1	T2
GY-PH	18	12.2	36.0	13.8	36.5
GY-HD	93	52.5	59.6	59.3	60.6
GY-TGW	28	22.0	9.3	24.8	9.7
GY-TW	116	52.5	60.4	59.4	62.6
GY-GPE	70	49.5	36.2	55.9	44.3
GY-EW	114	58.1	44.6	65.7	68.1

[a] R^2_{Total} was computed by fitting all *N* associated markers together in different multiple-regression models according to each trait pair (T1-T2).

Table S11. Power of the test developed by Jiang and Zeng (1995) to differentiate close linkage from pleiotropy in linkage simulated scenarios considering different levels of minor allele frequency (MAF), unbalanced percentage of explained genetic variation (QTL size) for each of the two simulated traits (T1 and T2), linkage disequilibrium (r^2) and marker profiles of the 358 European winter plus 15 spring wheat varieties population (GABI-WHEAT population). Each value corresponds to the proportion of times in which $H_0: p(1) = p(2)$ was rejected in 100 simulated replicates.

	QTL size		r^2		
			~0.55	~0.70	~0.91
MAF	T1	T2	(0.53-0.56)	(0.68-0.72)	(0.90-0.93)
	15	10	0.34	0.14	0.05
~0.06	15	5	0.11	0.06	0.07
(0.06-0.07)	10	15	0.21	0.16	0.04
	5	15	0.08	0.08	0.03
	15	10	0.39	0.32	0.15
~0.13	15	5	0.25	0.16	0.10
(0.11-0.14)	10	15	0.40	0.25	0.18
	5	15	0.22	0.23	0.10
	15	10	0.37	0.32	0.19
~0.22	15	5	0.36	0.32	0.06
(0.20-0.24)	10	15	0.48	0.43	0.16
	5	15	0.44	0.33	0.12
	15	10	0.56	0.45	0.19
~0.46	15	5	0.41	0.24	0.12
(0.44-0.48)	10	15	0.48	0.36	0.33
	5	15	0.45	0.43	0.18

Table S12. Type I error rate of the test developed by Jiang and Zeng (1995) under pleiotropy simulated conditions considering different levels of minor allele frequency (MAF), balanced along with unbalanced percentage of explained genetic variation (QTL size) for each of the two simulated traits (T1 and T2) and marker information of the 358 European winter plus 15 spring wheat varieties population (GABI-WHEAT population). Each value corresponds to the proportion of times in which $H_0: p(1) = p(2)$ was wrongly rejected in 100 simulated replicates.

	QTL size		
MAF	T1	T2	Type I error rate
	15	15	0.02
	10	10	0.03
	5	5	0.02
0.07	15	10	0.04
	15	5	0.02
	10	15	0.01
	5	15	0.05
	15	15	0.05
	10	10	0.13
	5	5	0.06
0.13	15	10	0.07
	15	5	0.13
	10	15	0.05
	5	15	0.16
	15	15	0.12
	10	10	0.08
	5	5	0.05
0.24	15	10	0.07
	15	5	0.09
	10	15	0.06
	5	15	0.05
	15	15	0.05
	10	10	0.08
	5	5	0.02
0.45	15	10	0.08
	15	5	0.07
	10	15	0.07
	5	15	0.09

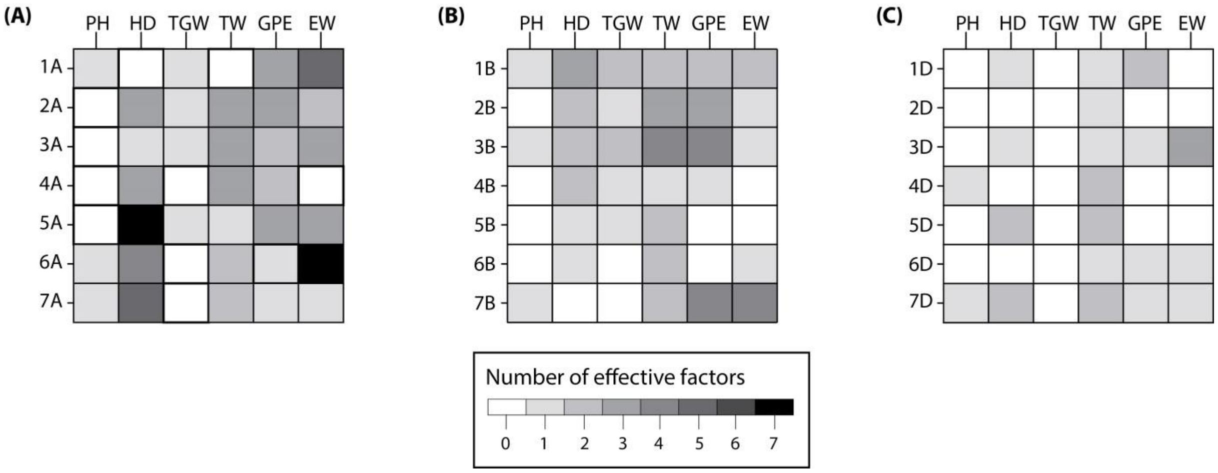

Fig. S1. Number of effective genetic factors by linkage-group being simultaneously associated (false discovery rate, FDR < 0.05) to grain yield (GY) and, at least, one GY-syndrome trait, as revealed by genome wide association scans (GWAS) using bivariate models in the population of 358 European winter plus 15 spring wheat varieties (GABI-WHEAT population) phenotyped in up to eight environments and genotyped with 18,856 polymorphic markers. Significant marker-trait associations were positioned according to past studies (Ellis *et al.*, 2002; Qin *et al.*, 2014; Wang *et al.* 2014) and grouped by genome: (A) A, (B) B and (C) D. GY-syndrome traits corresponded to plant height (PH), heading date (HD), thousand grains weight (TGW), test weight (TW), grains per ear (GPE), and ear weight (EW).

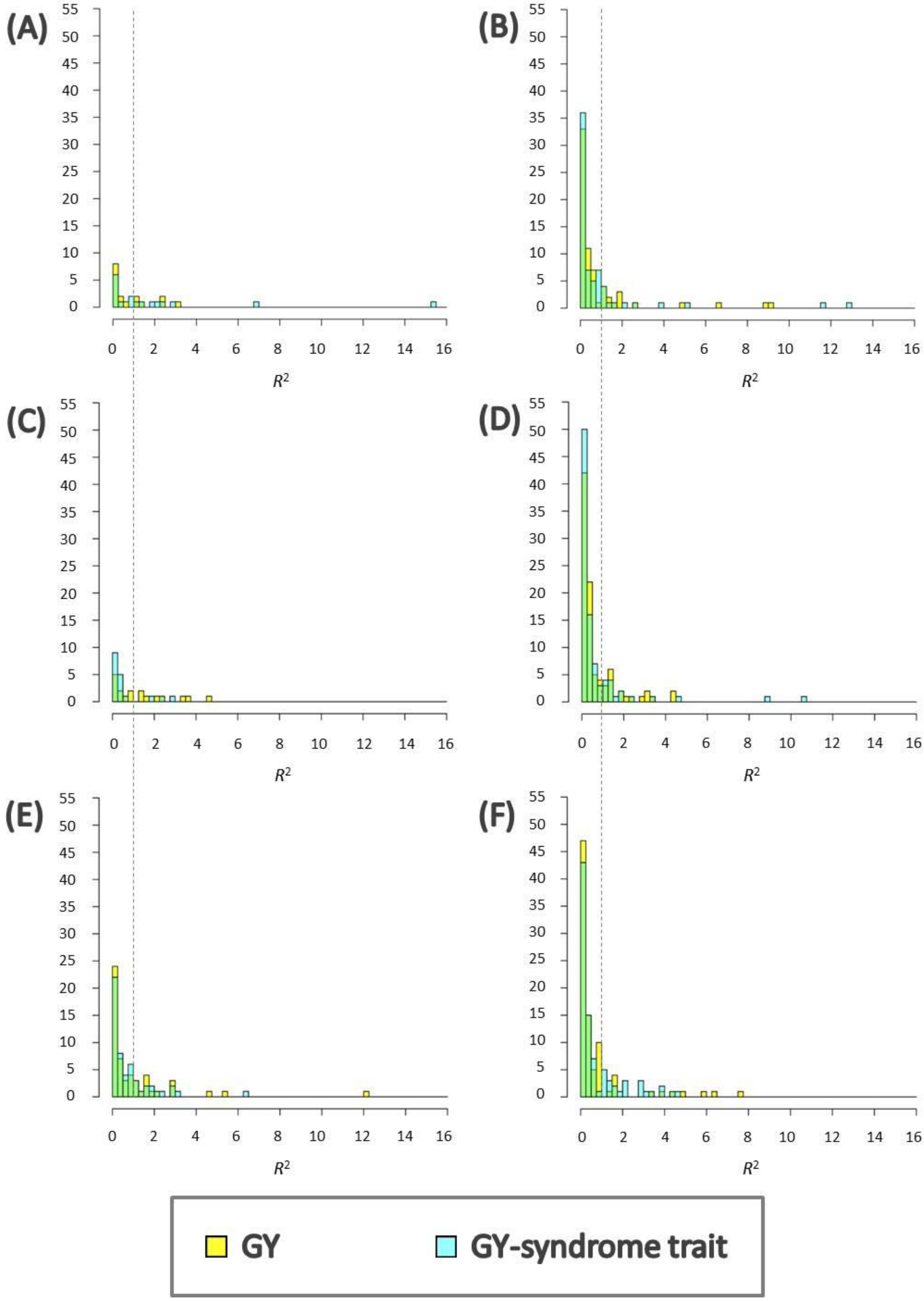

Fig. S2. Frequency distributions of explained genetic variance (R^2) of grain yield (GY) and GY-syndrome traits for markers simultaneously associated with GY and one of the following GY-syndrome traits: (A) plant height, (B) heading date, (C) thousand grains weight, (D) test weight, (E) grains per ear and (F) ear weight. The 1% R^2 threshold is indicated with a dashed line in graphs.

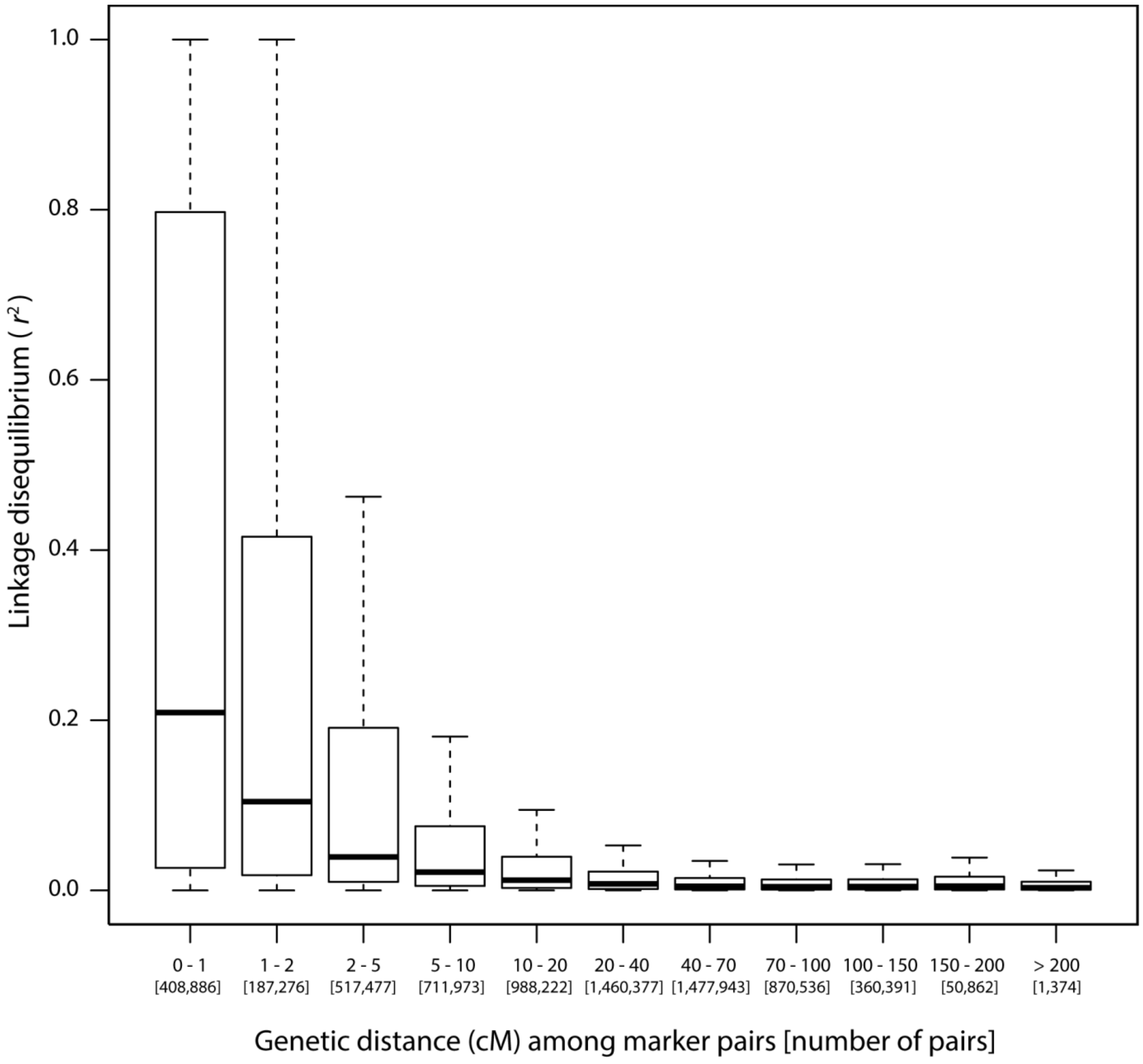

Fig. S3. Boxplot representing the decay of intra-chromosomal pairwise linkage disequilibrium (r^2) as a function of genetic distance (cM) between SNP markers in the population of 358 European winter plus 15 spring wheat varieties (GABI-WHEAT population). Genetic distances were calculated using genetic positions published by Wang *et al.* (2014).

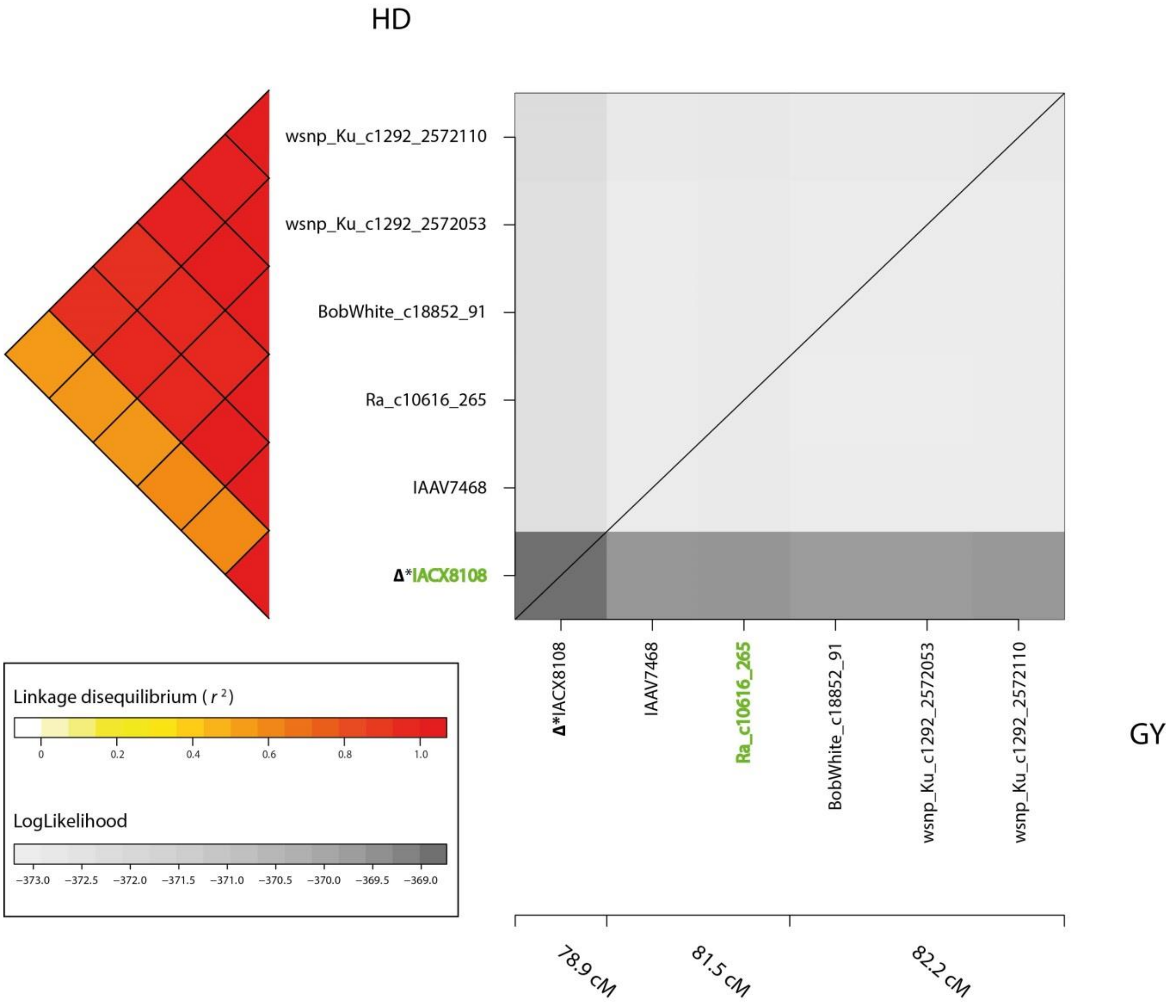

Fig. S4. Bivariate likelihoods (log-Likelihoods) during the two-dimensional scan to disentangle close linkage from pleiotropy in the surroundings of *IACX8108* (denoted with *), a marker simultaneously associated with grain yield (GY) and heading date (HD) at 78.9 cM on chromosome 2A (Fig. 1A; Table S5). Likelihoods pertaining pleiotropy models were maximized at marker *IACX8108* (indicated with Δ), whereas likelihoods for linkage models were maximized at the combination of *Ra_c10616_265* and *IACX8108* (highlighted in green); carrying these two last markers the effects on GY and HD, respectively. The log-likelihood ratio test of Jiang and Zeng (1995) using maximized likelihoods could not reject $H_0: p(1) = p(2)$ of pleiotropy at the nominal significance level of 0.05.

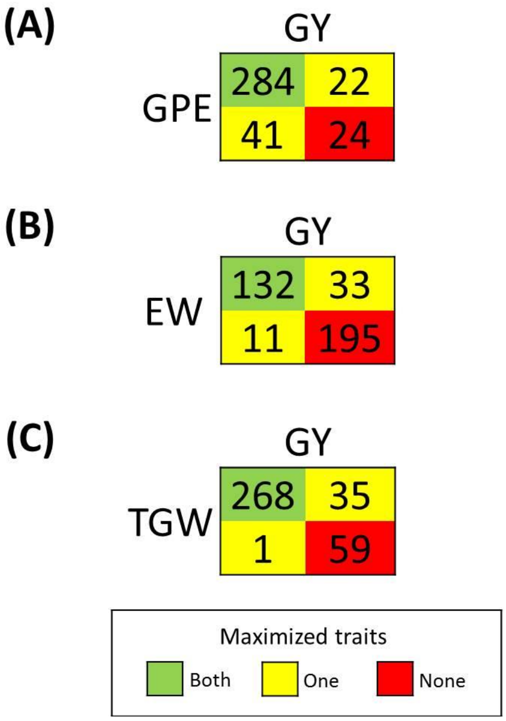

Fig. S5. Haplotype frequencies and their bivariate associations (maximizing both, one or none trait) for cases disentangled as close linkage by the log-likelihood ratio test of pleiotropy vs close linkage (Jiang and Zeng, 1995) in the population of 358 European winter plus 15 spring wheat varieties (GABI-WHEAT population). (A) Markers *wsnp_Ku_c16432_25320146* and *Tdurum_contig13240_523* carrying grain yield (GY) and grains per ear (GPE) quantitative trait loci (QTL), correspondingly. (B) Markers *Tdurum_contig30930_184* and *wsnp_JD_rep_c67103_42432235* associated to GY and ear weight (EW), respectively. (C) Markers *BS00098520_51* and *BS00003586_51* carrying effects on GY and thousand grains weight (TGW), correspondingly.

References

- **Beales J, Turner A, Griffiths S, Snape JW, Laurie DA.** 2007. A pseudo-response regulator is misexpressed in the photoperiod insensitive *Ppd-D1a* mutant of wheat (*Triticum aestivum* L.). Theoretical and Applied Genetics **115**, 721–733.
- **Butow BJ, Gale KR, Ikea J, Juhász A, Bedö Z, Tamás L, Gianibelli MC.** 2004. Dissemination of the highly expressed Bx7 glutenin subunit (*Glu-B1al* allele) in wheat as revealed by novel PCR markers and RP-HPLC. Theoretical and Applied Genetics **109**, 1525–1535.
- **Chartrain L, Brading PA, Brown JKM.** 2005. Presence of the *Stb6* gene for resistance to septoria tritici blotch (*Mycosphaerella graminicola*) in cultivars used in wheat-breeding programmes worldwide. Plant Pathology **54**, 134-143.
- **Ellis MH, Spielmeyer W, Gale KR, Rebetzke GJ, Richards RA.** 2002. 'Perfect' markers for the *Rht-B1b* and *Rht-D1b* dwarfing genes in wheat. Theoretical and Applied Genetics **105**, 1038-1042.
- **Gautier MF, Aleman ME, Guirao A, Marion D, Joudrier P.** 1994. *Triticum aestivum* puroindolines, two basic cystine-rich seed proteins: cDNA sequence analysis and developmental gene expression. Plant Molecular Biology **25**, 43-57.
- **Groenewald JZ, Marais AS, Marais GF.** 2003. Amplified fragment length polymorphism-derived microsatellite sequence linked to the *Pch1* and *Ep-D1* loci in common wheat. Plant Breeding **122**, 83–85.
- **Hanif M, Gao F, Liu J, Wen W, Zhang Y, Rasheed A, Xia X, He Z, Cao S.** 2016. *TaTGW6-A1*, an ortholog of rice *TGW6*, is associated with grain weight and yield in bread wheat. Molecular Breeding **36**, 1–8.
- **Hou J, Jiang Q, Hao C, Wang Y, Zhang H, Zhang X.** 2014. Global selection on sucrose synthase haplotypes during a century of wheat breeding. Plant Physiology **164**, 1918–1929.
- **Huang XQ, Röder MS.** 2005. Development of SNP assays for genotyping the puroindoline b gene for grain hardness in wheat using pyrosequencing. Journal of Agricultural and Food Chemistry **53**, 2070-2075.
- **Jiang Y, Jiang Q, Hao C, Hou J, Wang L, Zhang H, Zhang S, Chen X, Zhang X.** 2015. A yield-associated gene *TaCWI*, in wheat: its function, selection and evolution in global breeding revealed by haplotype analysis. Theoretical and Applied Genetics **128**, 131–143.
- **Jiang C, Zeng ZB.** 1995. Multiple trait analysis for genetic mapping of quantitative trait loci. Genetics **140**, 1111–1127.
- **Kollers S, Rodemann B, Ling J, Korzun V, Ebmeyer E, Argillier O, Hinze M, Plieske J, Kulosa D, Ganal MW, Röder MS.** 2013*a*. Whole genome association mapping of *Fusarium* head blight resistance in European winter wheat (*Triticum aestivum* L.). PLoS ONE **8**, e57500.
- **Kollers S, Rodemann B, Ling J, *et al.*** 2013*b*. Genetic architecture of resistance to Septoria tritici blotch (*Mycosphaerella graminicola*) in European winter wheat. Molecular Breeding **32**, 411–423.
- **Korzun V, Roder MS, Ganal MW, Worland AJ, Law CN.** 1998. Genetic analysis of the dwarfing gene *Rht8* in wheat. Part I. Molecular mapping of *Rht8* on the short arm of chromosome 2D of bread wheat (*Triticum aestivum* L.). Theoretical and Applied Genetics **96**, 1104–1109.
- **Liu S, Chao S, Anderson JA.** 2008. New DNA markers for high molecular weight glutenin subunits in wheat. Theoretical and Applied Genetics **118**, 177–183.
- **McMillin D, Allan RE, Roberts DE.** 1986. Association of an isozyme locus and strawbreaker foot rot resistance derived from *Aegilops ventricosa* in wheat. Theoretical and Applied Genetics **72**, 743–747.
- **Perovic D, Förster J, Devaux P, *et al.*** 2009. Mapping and diagnostic marker development for soil-borne cereal mosaic virus resistance in bread wheat. Molecular Breeding **23**, 641–653.
- **Qin L, Hao C, Hou J, Wang Y, Li T, Wang L, Ma Z, Zhang X.** 2014. Homologous haplotypes, expression, genetic effects and geographic distribution of the wheat yield gene *TaGW2*. BMC Plant Biology **14**, 107.
- **Rogers JS.** 1972. Measures of genetic similarity and genetic distance. Studies in genetics **7**, 145-153.
- **Searle SR.** 2006. Matrix algebra useful for statistics. 2nd Edition. Hoboken: Wiley-Interscience.

- **Schwarz G, Felsenstein FG, Wenzel G.** 2004. Development and validation of a PCR-based marker assay for negative selection of the HMW glutenin allele *Glu-B1-1d* (*Bx-6*) in wheat. Theoretical and Applied Genetics **109**, 1064–1069.
- **Su ZQ, Hao CY, Wang LF, Dong YC, Zhang XY.** 2011. Identification and development of a functional marker of *TaGW2* associated with grain weight in bread wheat (*Triticum aestivum* L.). Theoretical and Applied Genetics **122**, 211–223.
- **Wang S, Wong D, Forrest K, *et al.*** 2014. Characterization of polyploid wheat genomic diversity using a high-density 90 000 single nucleotide polymorphism array. Plant Biotechnology Journal **12**, 787–796.
- **Xia LQ, Ganal MW, Shewry PR, He ZH, Yang Y, Röder MS.** 2008. Exploiting the diversity of *Viviparous-1* gene associated with pre-harvest sprouting tolerance in European wheat varieties. Euphytica **159**, 411-417.
- **Zanke C, Ling J, Plieske J, *et al.*** 2015. Analysis of main effect QTL for thousand grain weight in European winter wheat (*Triticum aestivum* L.) by genome-wide association mapping. Frontiers in Plant Science **6**, 644.
- **Zhang Y, Liu J, Xia X, He Z.** 2014. *TaGS-D1*, an ortholog of rice *OsGS3*, is associated with grain weight and grain length in common wheat. Molecular Breeding **34**, 1097–1107.
